# Comparative analysis of placental transmission mechanisms for Dengue and Zika viruses: outcomes and future directions

**DOI:** 10.3389/fimmu.2026.1749309

**Published:** 2026-03-06

**Authors:** Indrani Mukherjee, Neil Ferland, Katelyn Tran Nguyen, Sara Moghaddam Adames, Helena Solo-Gabriele, Joshua Anzinger, Ivan A. Gonzalez, Ruby Dhar, Subhradip Karmakar, Rana Chakraborty

**Affiliations:** 1 Department of Pediatrics, Division of Infectious Diseases, University of Miami, Miami, FL, United States; 2 Department of Chemical, Environmental, and Materials Engineering, University of Miami, Coral Gables, FL, United States; 3 Department of Microbiology, The University of the West Indies, Kingston, Jamaica; 4 Department of Biochemistry, All India Institute of Medical Sciences, New Delhi, India

**Keywords:** Dengue, immunological factors, placenta, vertical transmission, Zika

## Abstract

Zika and Dengue viruses are arboviral pathogens capable of crossing the placental barrier, representing major global health risks for maternal and fetal outcomes. In this narrative review, we compare their epidemiology, clinical consequences in pregnancy, and underlying mechanisms of vertical transmission. Emerging molecular insights are highlighted, including disruptions to placental signaling pathways such as JAK/STAT and mTOR, and strategies to evade Hofbauer cells and the immune system. A comparative analysis of these processes underscores a critical need for improved understanding of placental pathophysiology, immune regulation, and molecular pathways of transmission. Identifying such mechanisms may promote vaccine development, improved diagnostics, and therapies to reduce adverse outcomes in the mother-infant dyad during maternal infection.

## Graphical Abstract

**Figure:**
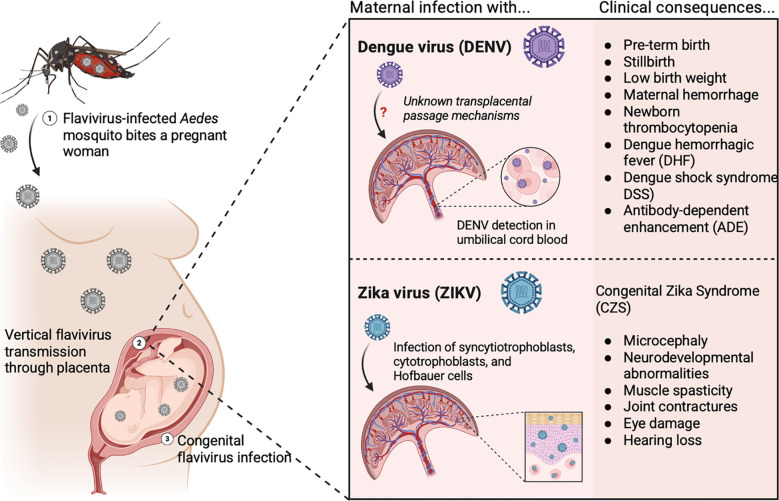
Vertical transmission of orthoflaviviruses (DENV or ZIKA) during pregnancy and associated clinical consequences. *Aedes* mosquitoes harboring DENV or ZIKV can infect a pregnant mother through a bite, which can be transmitted to the fetus via the placenta. DENV passes through the placenta through unknown mechanisms, but DENV has been detected in umbilical cord blood and can lead to pre-term birth and thrombocytopenia in the infant. ZIKV is known to infect syncytiotrophoblasts, cytotrophoblasts, and Hofbauer cells and can cause Congenital Zika Syndrome.

## 1 Introduction

The placenta functions as an immunological barrier protecting the developing fetus from pathogenic infections while simultaneously facilitating essential maternal-fetal exchange of nutrients, gases, and metabolic substrates through specialized syncytiotrophoblast (STB) and cytotrophoblast (CTB) populations, supported by a complex array of maternal decidual immune cells (1). However, certain viral pathogens have evolved sophisticated immune evasion mechanisms that enable placental cell infection and vertical transmission to the developing fetus (2). Trophoblast invasion occurs within a highly immunologically active maternal-fetal interface (MFI) characterized by a specific immune cell composition, which includes: 70% decidual natural killer (dNK) cells, 20-25% villous macrophages (Hofbauer cells), 3-10% T lymphocytes, and 1.7% dendritic cells, with decidual cytotoxic T cell and macrophage populations exhibiting dynamic fluctuations throughout gestation (3–6). The persistent presence of NK cells in decidual tissue during the first trimester and throughout pregnancy reflects their essential role in establishing immunotolerant conditions, while maintaining antimicrobial surveillance capabilities (7). Despite these robust immune defenses, Zika (ZIKV) and Dengue (DENV) and additional viruses such as human cytomegalovirus (HCMV) and HIV, have evolved to circumvent placental immune barriers, resulting in vertical transmission with fetal infection (8).

Both ZIKV and DENV are public health threats in tropical and subtropical regions and transmitted primarily through *Aedes* species mosquitos. While *Aedes aegypti* and *Aedes albopictus* are the most common vectors, other *Aedes* species such as *Aedes scutellaris*, *Aedes polynesiensis*, *Aedes malayensis*, and *Aedes mediovittatus* are known to be competent viral vectors for both ZIKV and DENV (9–12). Both viruses exhibit varying capacities for transplacental transmission associated with adverse pregnancy outcomes (13, 14). ZIKV exhibits well-documented tropism for placental cell populations, particularly in CTBs, STBs, and Hofbauer cells, while DENV has been detected in placental and fetal tissue (15–18).

ZIKV infection during pregnancy (19, 20), especially during the first trimester, is associated with congenital Zika syndrome (CZS), characterized by microcephaly, neurodevelopmental abnormalities, and fetal malformations (13, 14). These same clinical effects have been observed during ZIKV co-infection with HIV (21). Studies have documented transplacental transmission of ZIKV by identifying viral proteins and RNA in placental tissue at different stages of gestation, associated with an increase in placental abnormalities (22, 23). Maternal DENV infection is associated with preterm birth, low birth weight, but without an established pattern of congenital anomalies (24, 25). DENV nonstructural (NS) proteins have been detected in placental and umbilical cord cells (26). The global DENV burden increased 8-fold between 2000–2019 according to WHO data, with expanding geographic distribution into previously non-endemic regions including Europe and continental United States (27). A critical factor influencing orthoflavivirus placental transmission involves antibody-dependent enhancement (ADE), wherein pre-existing maternal antibodies from prior DENV infections paradoxically facilitate ZIKV cellular entry through an Fc receptor-mediated mechanism (28–30). Immunological cross-reactivity between DENV and ZIKV can potentially increase infectious susceptibility in pregnant women leading to adverse maternal-fetal health outcomes.

Given the escalating incidence of orthoflavivirus epidemics, comprehensive elucidation of transplacental transmission mechanisms represents a critical research priority to promote development of targeted therapies, prophylactic vaccines, and evidence-based public health interventions. This review provides a comparative evaluation of DENV and ZIKV placental cell tropism, maternal-fetal immunological interactions, clinical outcomes, and summarizes preventive approaches, while identifying substantial knowledge gaps and delineating future research priorities.

## 2 The general epidemiology of DENV and ZIKV infections

**Figure 1:**
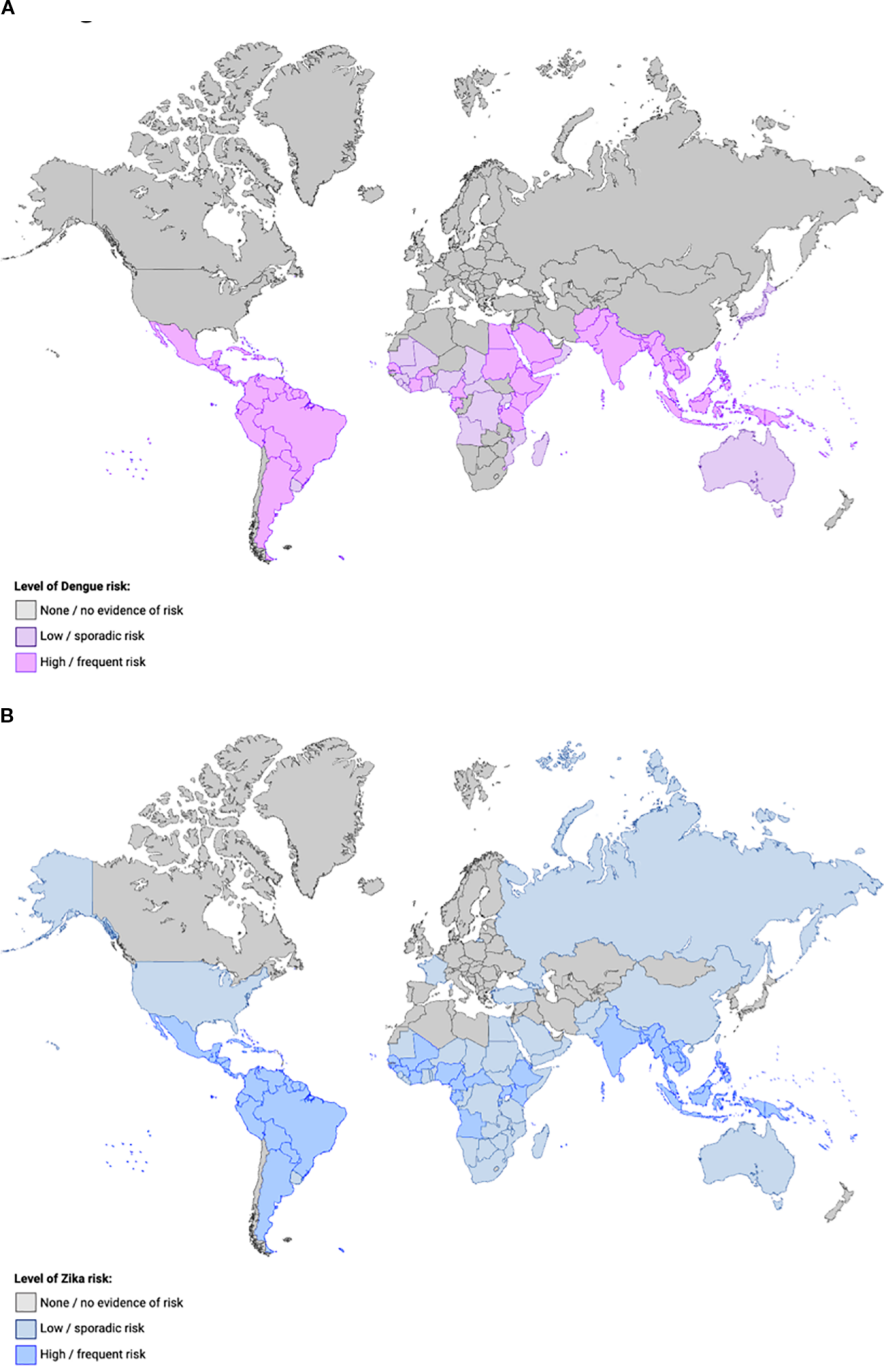
World map demonstrating the epidemiology of DENV **(A)** and ZIKV **(B)**. Countries are shaded according to the estimated level of transmission risk of DENV (**(A)** purple) or ZIKV (**(B)** blue) from 2025 CDC data (33, 34): grey indicates little or no evidence of risk (**(A)** no reported DENV cases in the past decade; **(B)** not known to have mosquitoes that transmit ZIKV), light colors indicate low or sporadic risk (**(A)** evidence of at least 1 locally acquired DENV case in the past decade; **(B)** known to have mosquitoes that transmit ZIKV but no ZIKV cases), and darker colors indicate high or frequent risk (**(A)** evidence of more than 10 locally acquired DENV cases in the past decade; **(B)** known to have mosquitoes that transmit ZIKV but no ZIKV cases).

DENV and ZIKV are single-stranded RNA arboviruses belonging to the Flaviviridae family in the genus *Orthoflavivirus* and are transmitted by *Aedes* mosquitoes (31). DENV was first isolated in 1943 in Japan and consists of four different serotypes (DENV1-4). The first isolated serotype was DENV-1, with DENV-2 being isolated two years later in Hawaii (32). After the first isolation, viral mapping showed that DENV was solely endemic to tropical and subtropical climates. However, as global travel became more common and climates changed, DENV infections spread to areas where the virus was not endemic. While all four serotypes were endemic to South America and Southeast Asia, cases were documented in continents with similar climates, such as Africa and Australia (**Figure 1A**). However, endemic transmission has also been reported in North America and Europe (32). ZIKV was first isolated from a rhesus monkey in the Zika Forest in Uganda in 1947, and for the first time outside of Africa in 1966 in Malaysia. Surveys conducted in the mid 1900’s suggested that this virus was widespread in both Africa and Asia before the time of discovery (35) (**Figure 1B**).

## 3 The clinical impact of arboviral infection during pregnancy

Throughout gestation, maternal physiology undergoes extensive immunological and physiological modifications to establish immune tolerance toward the semi-allogeneic fetus, which would ordinarily trigger alloimmune rejection responses from paternal antigenic components. These immune adaptations, essential for successful fetal accommodation and development, compromise maternal immunocompetence and increase susceptibility to viral pathogens (36). This section summarizes the clinical consequences of maternal DENV, ZIKV, or coinfection in pregnancy.

### 3.1 Dengue viral infection in pregnancy is associated with dengue hemorrhagic fever, dengue shock syndrome, stillbirth, and preterm birth

DENV seroprevalence among pregnant women in endemic areas often surpasses 50%, although diagnostic challenges, particularly in the early phase of infection and during co-infection, hinder appropriate risk assessment and response (37). The clinical manifestations of DENV range from asymptomatic or mild flu-like symptoms to organ failure (38). In pregnant women, DENV infection can be more marked with an increased risk of stillbirth (36). The clinical manifestations of severe maternal dengue include dengue hemorrhagic fever (DHF) and dengue shock syndrome (DSS). Both conditions increase the likelihood of maternal ICU admissions and obstetric interventions (39). Recent systematic reviews and two meta-analyses projected a preterm birth risk of 18–25% and a stillbirth risk at 5–7%, with greater severity associated with high viral load (40, 41). These findings, considered together, underscore an urgent need for gestational surveillance, vector control, improved diagnosis, and maternal immunization strategies to reduce the impact of DENV during pregnancy.

### 3.2 Megakaryopoiesis disruption and thrombocytopenia during DENV infection

**Figure 2:**
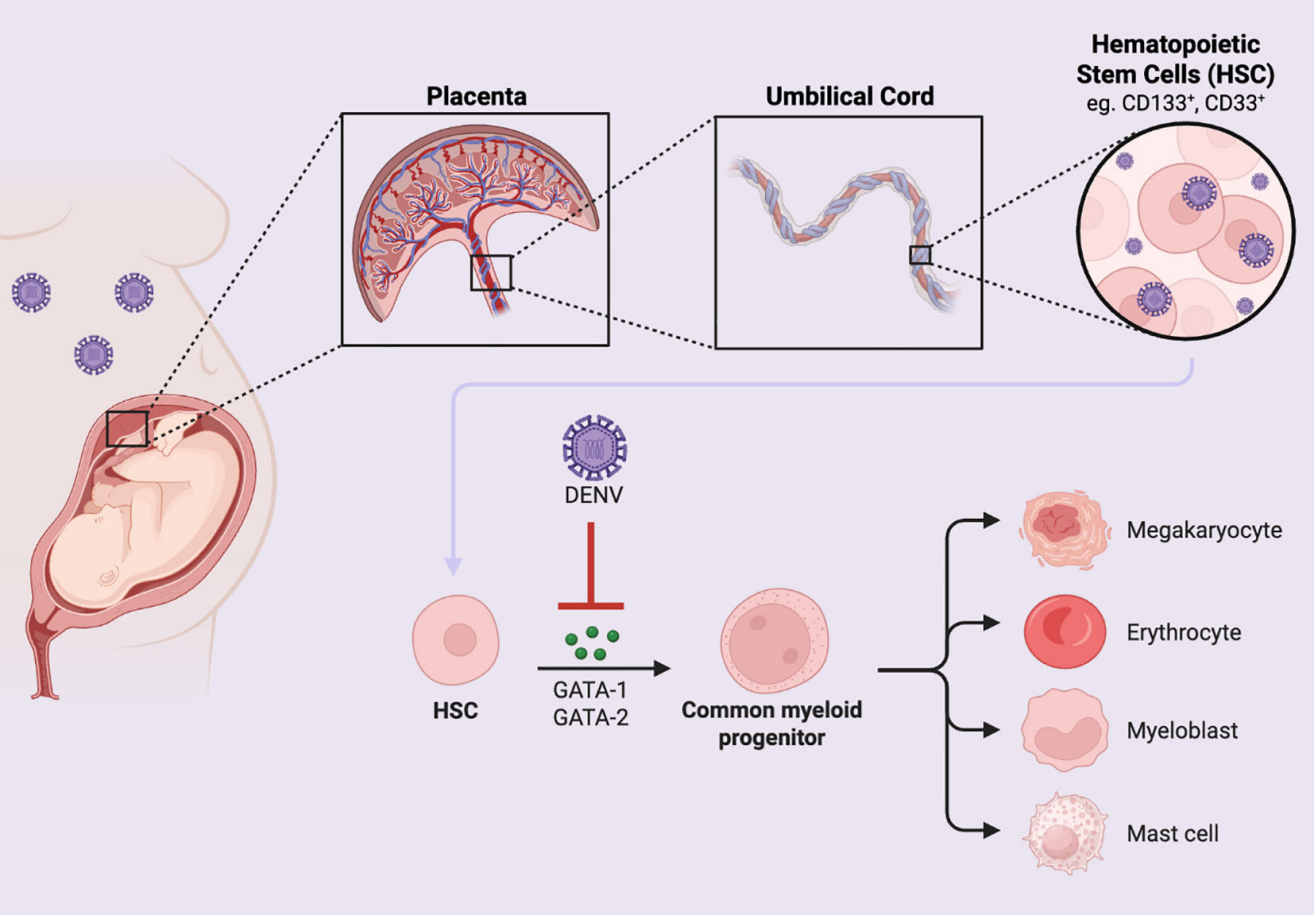
DENV downregulates hematopoiesis during perinatal infection. DENV can be detected in the umbilical cord of a DENV-infected pregnant person. DENV can impair the differentiation of HSCs into myeloid lineages through inhibition of the transcription factors, GATA-1 and GATA-2.

The main molecular pathway impacted by DENV infection is the PI3K/AKT/mTOR pathway, which plays a role in cell survival, maturation, and megakaryocyte development (42). The activation of PI3K and AKT regulates platelet production in megakaryocytes and promotes cell survival by inhibiting apoptosis. Several transcription factors are involved in platelet production by megakaryocytes. These include GATA-1 and GATA-2, which regulate polyploidization, cell cycle progression, and expression of genes specific to megakaryopoiesis through the downstream effector, STAT1. The other is NF-E2, which is required for megakaryocyte development and maturation, and for determining the stages of megakaryopoiesis (42). DENV infection in megakaryocyte cell lineages impairs AKT activation by first impacting PI3K, an upstream signaling molecule of AKT, reducing the expression of both regulator (p85) and catalytic units (p110β). DENV infection also results in decreased activation of the downstream effector proteins P70-S6 kinase and PKC-α, which affects the function of mTORC1 and mTORC2. The downstream impact of DENV infection on signaling and effector proteins in this pathway prevents expression of GATA-1, GATA-2, and NF-E2 transcription factors, impairing megakaryopoiesis (**Figure 2**). DENV infection also decreases expression of the anti-apoptotic protein Bcl-2, providing further evidence that dengue can stimulate apoptosis in megakaryotes (42). These mechanism may explain how DENV causes thrombocytopenia, DHF, and DSS in infected individuals.

### 3.3 Infant outcomes of DENV infection in pregnancy include low birth weight, and intrauterine growth restriction

Recent DENV outbreaks from Brazil, Southeast Asia, and India document that symptomatic maternal DENV in pregnancy is associated with intrauterine growth restriction (IUGR), low birth weight, and newborn thrombocytopenia (41, 43, 44). The evidence demonstrating the ability of DENV to be vertically transmitted from mother to baby during pregnancy is not as robustly supported as it is for ZIKV. However, rare cases document DENV in neonates only days after birth, in the absence of mosquito bites (45, 46). In these cases of vertical transmission when maternal DENV infection was documented peripartum, DENV RNA was detected in cord blood, placental tissue, and infant serum (47). Other studies have provided additional circumstantial evidence to support vertical transmission by detecting dengue NS proteins in the neonatal serum of newborns (48–50). Maternal anti-DENV antibodies could influence newborn immunological outcomes; in rare cases, ADE has been proposed as a mechanism for worsening maternal or infant illness (51).

### 3.4 Diagnostic challenges to neonatal dengue recognition in postnatal life

A comprehensive case series involving 32 neonates with confirmed DENV infection, the largest and only multicenter neonatal dengue case study reported to date, demonstrated the substantial prevalence of diagnostic errors in neonatal dengue recognition, with 25% of cases initially misdiagnosed as neonatal sepsis and 12.5% erroneously attributed to immune thrombocytopenia (52). The mean symptom onset occurred at 7 days postpartum, creating an extended temporal window for misdiagnosis and inappropriate therapeutic interventions. Although no neonates developed severe dengue manifestations, the clinical presentation included petechiae in 87.5% of cases, hepatomegaly in 75%, and pharyngeal haemorrhage in two patients. This report underscores both the frequency of diagnostic misattribution and the diagnostic challenges associated with neonatal dengue. Furthermore, serological profiles varied significantly with some neonates testing positive for both NS1 antigen and IgM antibodies while others exhibited isolated marker positivity. In addition, nearly 20% of confirmed cases were asymptomatic despite virological confirmation. A case study conducted between October-November 2021 during a DENV epidemic at a single hospital in Pakistan (53) documented five neonates presenting with clinical symptoms of neonatal sepsis. All five tested positive for NS1, three were diagnosed with DENV fever, one with DHF, and the other with DSS. These reports highlight how neonatal DENV infection cryptically mimics neonatal sepsis, underscoring the need to incorporate routine consideration of DENV infection in neonates presenting with sepsis-like symptoms in areas and timeframes when DENV is known to be endemic.

### 3.5 The clinical manifestations Congenital Zika Syndrome

In contrast to DENV, ZIKV infection during pregnancy can result in Congenital Zika Syndrome (CZS). The teratogenic potential of ZIKV especially in the first trimester, was documented by an outbreak in Brazil with subsequent spread throughout the Americas between 2015 and 2017 (54). Latent viral circulation and underdiagnosis in asymptomatic individuals made surveillance and prompt intervention challenging (55). Regional differences and the need for improved prenatal screening were highlighted by a major retrospective study conducted in French Guiana that reported an incidence of ZIKV infection in pregnancy as high as 188 per 1,000 (56). The impact of early detection and vector control were underscored by cohort data from investigators in Northeastern Thailand and Brazil, who noted that even mild or asymptomatic maternal infection resulted in adverse neonatal outcomes (57, 58).

ZIKV has a particular capacity among orthoflaviviruses to cross the placenta and target fetal neural progenitor cells. Cohort studies from Brazil, Colombia, and Puerto Rico revealed a spectrum of clinical manifestations ranging from fetal loss to severe neurological disability in postnatal life. Specific clinical manifestations include fetal brain disruption sequence (59) characterized by microcephaly, cerebral calcifications, structural brain abnormalities, and neurodevelopmental delay. Other manifestations of CZS include ocular abnormalities, and limb and joint contractures (60, 61). Though risks are present throughout gestation, studies show that first-trimester infection during embryogenesis is strongly related to adverse fetal outcomes. ZIKV RNA has been detected in amniotic fluid, and fetal brain tissue (62, 63). ZIKV and DENV circulate simultaneously in many regions. By utilizing ADE, maternal DENV immunity could promote ZIKV proliferation exacerbating placental damage (64–66).

### 3.6 Neonatal ZIKV transmission and the impact on infant neurodevelopment

Neonatal ZIKV acquisition occurs through three primary transmission routes: transplacental vertical transmission during gestation, perinatal exposure during the intrapartum and immediate postpartum periods, and postnatal acquisition including breastfeeding-mediated transfer (67). While CZS has garnered significant attention, contemporary studies demonstrate that neonates with *in-utero* ZIKV exposure exhibit ongoing neuroinflammatory processes and progressive synaptic dysfunction that extend beyond the classical CZS phenotype.

The persistent neuropathological manifestations of ZIKV in neonatal populations can manifest as a heterogenous spectrum of persistent neurodevelopmental and neurologic outcomes. Benazzato et al. (68) demonstrated through analysis of brain tissue specimens from infants with CZS that vertical ZIKV transmission induces sustained neuroinflammatory responses that disrupt synaptic function. By extrapolation, these data provide evidence for the long-term neurological consequences of perinatal ZIKV exposure and underscore the need for continuous neurodevelopmental and neurological monitoring for affected neonates including those who appear asymptomatic at birth. In addition, recent studies have evaluated neurological and ophthalmologic symptoms in newborns exposed to ZIKV. According to a case report from Puerto Rico, a premature baby was later discovered to have ocular defects linked to ZIKV after being initially misdiagnosed with a different congenital condition (69, 70). This report highlights the ongoing need for clinicians, particularly in endemic areas and for individuals with travel history to affected regions, to consider ZIKV in the differential diagnosis when neonates exhibit unexplained developmental or neurological abnormalities.

### 3.7 The association of Dengue and Zika virus infections with Guillain Barre syndrome

There is a strong association between ZIKV and DENV infection and the development of Guillain-Barré syndrome (GBS). GBS is an immune-mediated disease of the peripheral nerves (71) and the most common cause of acute neuromuscular paralysis (72). A case-control study conducted in Puerto Rico in 2016 during a ZIKV epidemic estimated the risk association between ZIKV infection and GBS development (73). There were 47 case-patients enrolled, and 39 had a confirmed diagnosis of GBS (83%). One of the risk factors identified for GBS development was acute ZIKV infection within two months prior to GBS onset, which occurred in 23% of the cases (73). Similar results were noted in a study of 68 GBS patients in Colombia in 2015–2016 during a ZIKV outbreak (74). Sixty-six patients had symptoms of ZIKV infection prior to GBS onset, with the median period between ZIKV symptoms and GBS onset being 7 days. 42 of these 66 individuals (64%) were positive for ZIKV by RT-PCR. While these studies were limited by small sample sizes, they demonstrated an association between ZIKV infection and the onset of GBS (74). An association between DENV infection and GBS has been documented but is less common than with ZIKV (75).

## 4 The molecular impact of arboviral infections on the placenta

To fully understand the impact that arboviruses have on the placenta during pregnancy, it is important to focus on the underlying molecular mechanisms and immune pathways impacted by these viruses. Here, we discuss key cellular pathways associated with DENV and ZIKV infection in pregnancy and how altered pathways correlate with clinical manifestations in neonates following maternal infection.

Vertical transmission represents critical windows for infectious disease acquisition in fetuses and infants (76). Maternal DENV infection induces increased vascular permeability and endothelial dysfunction, compromising placental integrity and facilitating vertical transmission at documented rates ranging from 1.6% to 22.7% (50). ZIKV vertical transmission rates is gestational age-dependent with some studies reporting mean transmission risks as high as 47% influenced by the trimester of maternal infection (77).

### 4.1 The impact of maternal Dengue Virus infection at the maternal fetal interface

Although the precise mechanisms by which DENV affects placental tissue at the cellular level remain poorly characterized, elucidating viral-induced alterations in cell signaling pathways that contribute to characteristic clinical manifestations is essential for understanding the impact of maternal infection on fetal and neonatal outcomes (78). In severe DENV, a life-threatening pathophysiological mechanism involves enhanced vascular permeability resulting from NS1 viral protein interactions with toll-like receptor (TLR) signaling pathways, causing endothelial tissue damage that precipitates plasma extravasation, systemic hypotension, circulatory collapse, and multi-organ dysfunction (78, 79).

Studies directly examining the impact of DENV infection at the MFI, in contrast, are limited. DENV has been detected in umbilical cord blood (UCB) cells and alters cytokine expression with increased production of IL-6 and IL-8 (80). Increased levels of these cytokines were also detected in the serum of patients diagnosed with DHF. These data suggest HUVECs may act as a target for DENV infection (80). Similar studies in human brain microvascular endothelial cells (HBMECs) demonstrate that DENV can infect and replicate in these cells, while increasing the secretion of the inflammatory cytokines IL-6 and IL-8, associated with an increase in reactive oxygen species (ROS) (81). The induction of ROS that occurs in HBMECs can be extrapolated to placental cells, providing a potential explanation for the increased rates of preeclampsia documented in pregnant women infected with DENV (82, 83). *In vitro* studies have noted the ability of DENV to infect CD133+ and CD34+ cells, potentially acting as replicative reservoirs (84). Murine studies have demonstrated that DENV can cross the placental barrier and be vertically transmitted under conditions that mimic ADE, providing further, albeit limited, evidence of the vertical transmission of DENV (85). An important aspect of maternal DENV infection in pregnancy is placental damage. Histopathological analyses have documented hypoxia, choriodeciduitis, deciduitis, and intervillositis in DENV-infected placentae (86). Additionally, murine studies have noted that DENV infection can lead to neutrophil infiltration, disrupting the vascular network (87) associated with adverse neonatal and maternal outcomes (88). Comparisons between DENV infection in the placenta can be drawn with SARS-CoV-2. Several studies have identified viral proteins of SARS-CoV-2 and active replication of the virus *in-vivo* in trophoblast, Hofbauer, and endothelial cells (89–91). The resulting inflammation can result in placental damage (92, 93). Despite placental inflammation, evidence for vertical transmission of SARS-CoV-2, like DENV, is limited (84, 94).

### 4.2 Molecular effects of ZIKV at the placenta that promote perinatal transmission and adversely impact synaptic development

**Figure 3:**
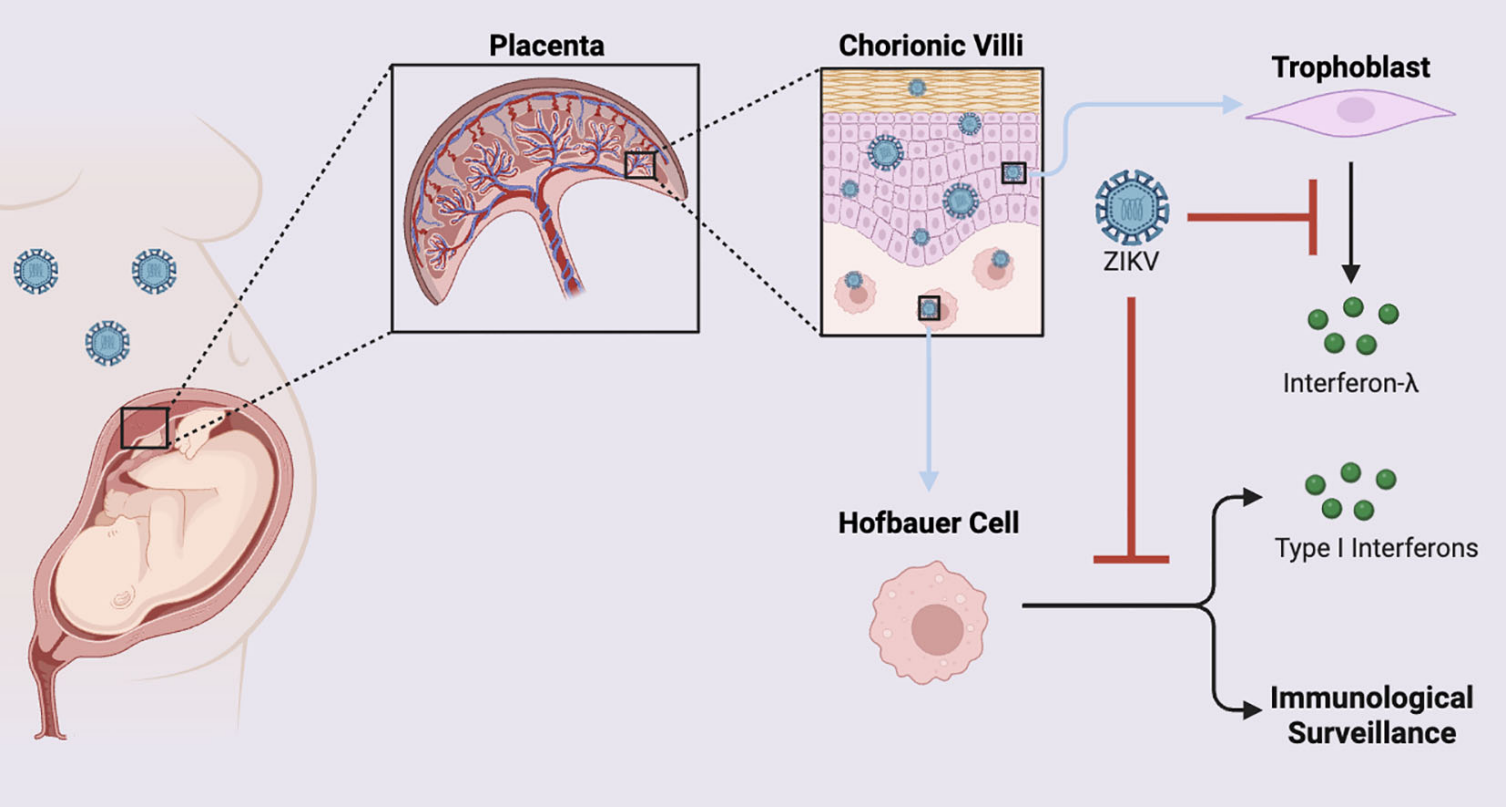
ZIKV evasion of placental trophoblast- and Hofbauer cell-mediated immune defenses during perinatal infection. Perinatal ZIKV infection of the chorionic villi can prevent trophoblasts lining the placenta from secreting IFN-λ, allowing for viral invasion. Furthermore, ZIKV can dampen the ability of Hofbauer cells to provide immune surveillance and type I IFN secretion, weakening the placental innate immune defenses and facilitating vertical transmission to the fetus.

**Figure 4:**
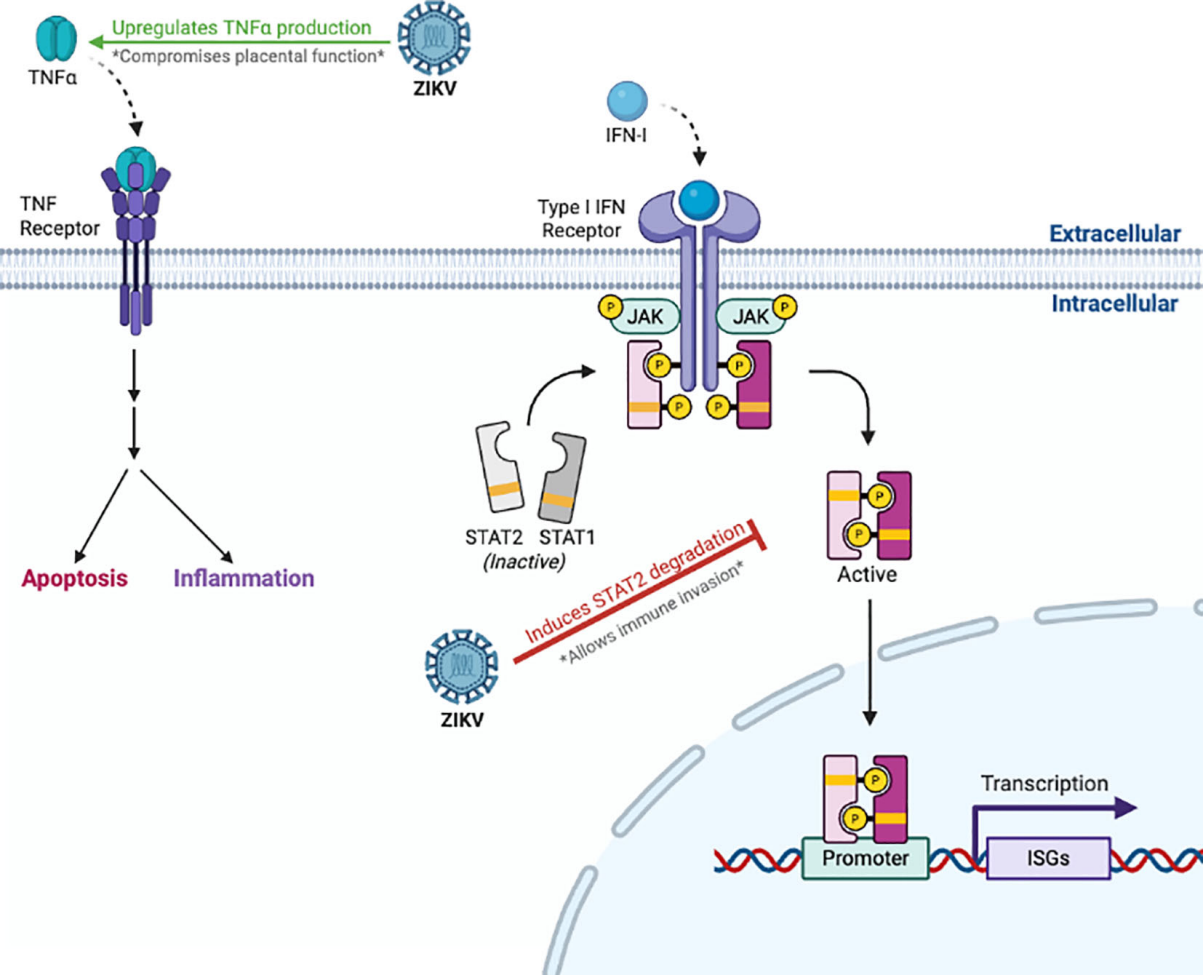
Perturbation of host immunity by ZIKV. ZIKV antagonizes IFN-I signaling by targeting STAT2 for proteosomal degradation, suppressing the transcription of ISGs and weakening antiviral defenses. ZIKV also compromises placental function by upregulating TNF-α production, leading to hyperinflammation and apoptosis.

Maternal and fetal immune responses interact intricately to affect ZIKV’s capacity to penetrate the placenta and infect the growing fetus. The placenta provides an immunological and physical barrier against viral infections. Trophoblasts generate type III interferons (IFN-λ), which aid in thwarting viral invasion (15). ZIKV can infect placental macrophages or Hofbauer cells, which provide an immunological barrier and are involved in immune surveillance. Upon infection, these cells promote viral dissemination throughout the placenta (68). In addition, ZIKV reduces the antiviral effects of type I IFN signaling by suppressing maternal IFN using the ZIKV NS5 protein, which targets the STAT2 host immune defense complex for degradation (95, 96) (**Figures 3**, 4) This method of immune evasion increases the risk of vertical transmission by enabling the virus to infiltrate the placenta.

Adverse gestational outcomes following maternal ZIKV infection are mechanistically linked to maternal immune activation, characterized by upregulation of pro-inflammatory cytokines including tumor necrosis factor-alpha (TNF-α) and IL-6. This has been shown to compromise placental physiological function and induce fetal neuroinflammatory responses (68) that adversely shape neurotranscriptional expression negatively influencing synaptic development (68, 97). This inflammatory cascade also enhances placental barrier permeability leading to cerebral edema, complicated further by placental ischemia (98). Infected placental cells release extracellular vesicles containing inflammatory mediators and viral ribonucleic acid, which can traverse to fetal organs and trigger neuroinflammatory cascades. This inflammation-mediated tissue damage may contribute to fetal brain disruption sequence and the severe neurological abnormalities characteristic of CZS (68).

### 4.3 Inhibition of trophoblast syncytialization by ZIKV infection

Trophoblast syncytialization is key to placental development, as these cells differentiate to form multinucleated syncytiotrophoblasts (STBs) that form the outer layer of the placenta (99). STBs are resistant to ZIKV infection, allowing them to serve as a barrier to transplacental spread (100). However, ZIKV can modulate this protective layer. Studies of ZIKV-infected placental tissue examined changes in paracellular permeability at the STB layer, normally controlled by tight junctions. The expression of tight junction proteins, such as E-cadherin, claudins, and occludin, were measured in addition to paracellular permeability (101). Tight junction protein expression was the same, apart from claudin-4, which was decreased. Additionally, the paracellular permeability of ZIKV-infected placentae was greater than in healthy control samples, suggesting ZIKV can modulate tight junction proteins, allowing for viral spread in the placenta (101).

Zika virus has been shown to prevent the differentiation of trophoblastic stem cells to syncytiotrophoblasts (102). An *in-vitro* study using placental organoids examined the vulnerability of human trophoblast stem cells to ZIKV infection. Trophoblasts were highly permissive to ZIKV, and resistance to infection increased as the stem cells began differentiating. Expression of *AXL* and *TIM-1* by trophoblast stem cells contributed to the sensitivity of these cells to ZIKV infection. *AXL* encodes a receptor tyrosine kinase of the TAM receptor family, while *TIM-1* produces a phosphatidylserine receptor, both functioning to facilitate ZIKV entry (102–105). The study concluded that ZIKV infection in trophoblast stem cells disrupts differentiation to cytotrophoblasts and syncytialization, providing an explanation for the placental damage often observed during maternal ZIKV infection (102).

### 4.4 ZIKV-mediated teratogenesis through endoplasmic reticulum stress induction

**Figure 5:**
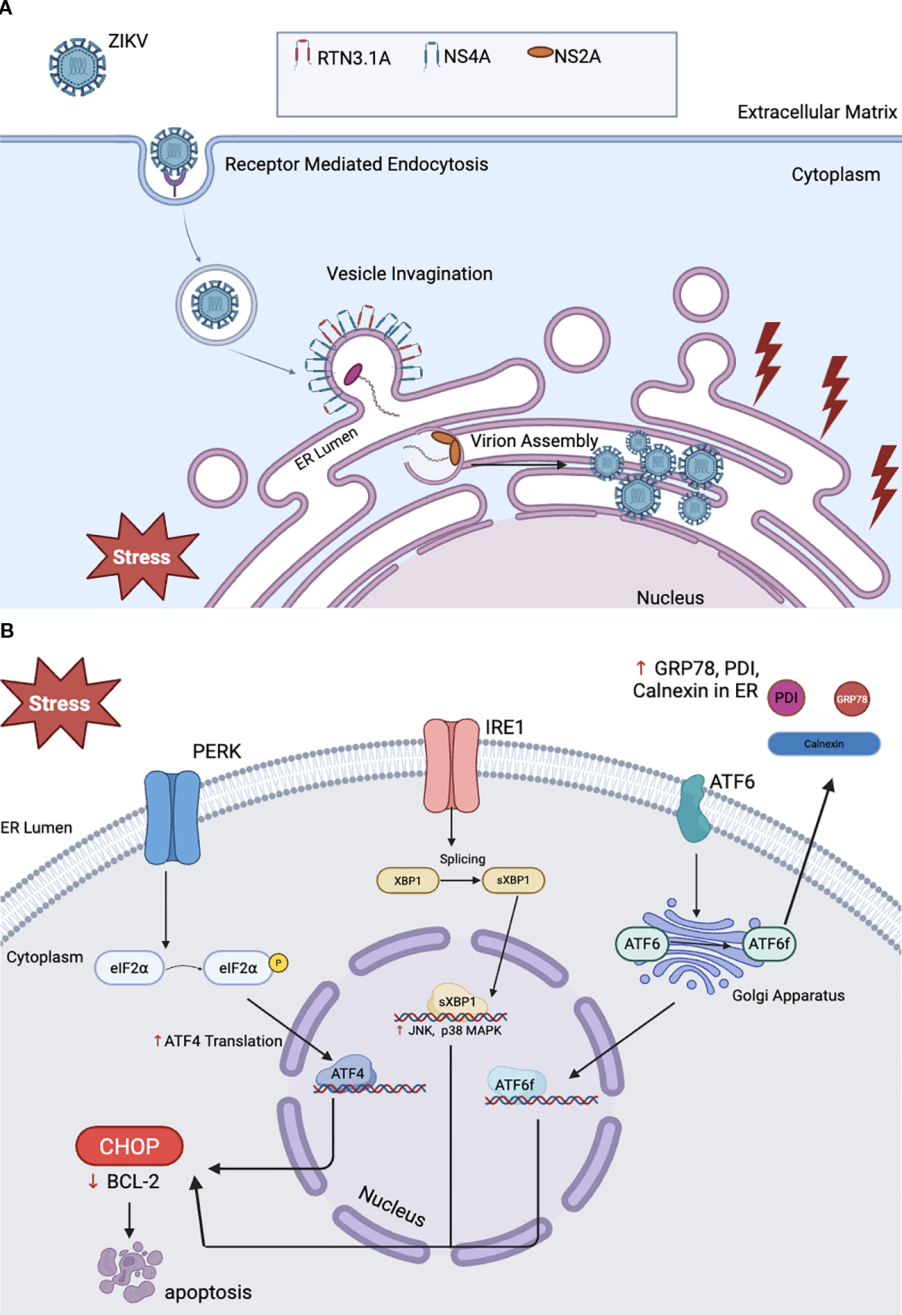
ZIKV-mediated teratogenesis through endoplasmic reticulum stress induction. **(A)** ZIKV enters the host cell via receptor-mediated endocytosis, using proteins NS4A and NS2A to remodel the ER membrane and create replication organelles for virion assembly. The reorganization of the ER membrane disrupts normal ER protein-folding capacity, triggering ER stress and activation of UPR. **(B)** Within the UPR, the PERK, IRE1, and ATF6 branches activate stress effectors, including increased translation of ATF4, splicing of XBP1 with subsequent activation of death proteins JNK and p38 MAPK, and expression of ATF6f. This culminates in CHOP upregulation, suppression of BCL-2, and apoptosis of placental and neural progenitor cells.

ZIKV can disrupt embryogenesis by triggering endoplasmic reticulum (ER) stress in both placental trophoblast populations and developing neural tissue (106, 107). Following receptor-mediated endocytosis, ZIKV gains entry into host cells and localizes to the ER, where viral genome replication and polyprotein translation occur (108). Upon cellular invasion, ZIKV exploits viral non-structural proteins NS4A and NS2A to commandeer ER membrane architecture, establishing specialized replication organelles including convoluted membrane structures (CM) and zippered ER formations (zER), that provide protected microenvironments for viral particle assembly and maturation (109, 110). The NS4A protein specifically binds host reticulon 3.1A (RTN3.1A) to orchestrate ER membrane remodeling, while NS2A facilitates viral ribonucleoprotein complex formation and recruits structural proteins to assembly sites through homotypic protein-protein interactions (109, 111) (**Figure 5A**). This extensive membrane reorganization exceeds the ER’s homeostatic protein folding capacity, precipitating ER stress conditions that activate the unfolded protein response (UPR) pathway (108, 109). The UPR activates C/EBP Homologous Protein (CHOP), leading to cell apoptosis. ZIKV achieves this by activating one of 3 UPR pathways: PERK, IRE1, and ATF6. Through PERK, ZIKV facilitates the phosphorylation of eIF2α to suppress protein synthesis; however, mRNAs like ATF4 become translated, activating CHOP (109–111) (**Figure 5B**). The IRE1 pathway splices XBP1 mRNA into its active form, XBP1s, and activates death proteins JNK and p38 MAPK, further promoting CHOP expression in the nucleus (106, 111, 112). ZIKV also triggers the cleavage of ATF6 to its active form (ATF6f) in the Golgi apparatus, allowing fragments to enter the nucleus and upregulate GRP78, PDI, and calnexin. These proteins reduce ER stress and, if unsuccessful, ultimately activate CHOP (108). After upregulation, CHOP functions as an apoptotic switch by suppressing the anti-apoptotic protein BCL-2. In fetal tissue, CHOP expression can result in apoptosis to neural progenitor cells, further contributing to fetal brain disruption sequence, when infection occurs in the first or second trimester (110). ZIKV persistence in trophoblast cells increases vertical transmission to the fetus (106, 107).

## 5 Systemic innate and adaptive host immune factors to arboviral infection

### 5.1 Acquired immunity to DENV and antibody-dependent enhancement

The innate immune response to DENV infection involves dendritic cells (DCs), macrophages, and monocytes as the initial responders to viral pathogens. The innate immune response to DENV is typical for any foreign pathogen: Pathogen recognition receptors (PRRs) on innate immune cells recognize pathogen-associated molecular patterns (PAMPs), and toll-like receptor (TLR) signaling triggers the production of inflammatory cytokines and IFNs (113). During pregnancy, the innate immune response to DENV changes. *In-vitro* studies in HTR8 cells, a human extravillous trophoblast cell line, found that DENV infection documented increased production of IL-6, IL-8, TNF-α, and CCL2 compared to non-infected cells (114). This increase in inflammatory cytokines is consistent with the characteristic cytokine storm of DENV.

Acquired immunity to DENV is mediated through the DENV neutralizing antibody responses to multiple serotypes in which infection with one results in long term protection but not against other serotypes. Neutralizing antibodies are commonly detected in individuals from countries with endemic dengue. These maternally derived neutralizing anti-DENV IgG antibodies efficiently cross the placental barrier may confer dengue immunity to newborns during the first few months of life (84, 115). Antibody titers for all four different DENV serotypes are known to be high initially after birth but steadily decay until they are absent after about one year (116). However, these antibodies can trigger an inflammatory response to DENV infection, where pathogens or infected cells bound by IgG form immune complexes and help neutralize DENV (117). One of the primary causes of fetal and neonatal death from DENV infection during pregnancy is insufficient maternally-derived IgG neutralizing antibodies (84). Most cases of DHF in infants occur when there are sub-optimal levels of maternally-derived neutralizing antibodies (117, 118).

However, non-neutralizing IgG can also be acquired after recurrent infections in mothers and in primary infection in infants (117). Unlike neutralizing antibodies, these are reactive and can lead to ADE. During ADE, non-neutralizing antibodies enhance pathogenesis through Fc gamma receptor (FcγR) mediated entry of the virus into leukocytes (119). These surface receptors recognize the Fc domain on antibodies (119), which triggers effector cells to respond during viral infection once activated. The normal pathway triggered by FcγR’s starts when the IgG immune complex binds, which triggers the phosphorylation of immunoreceptor tyrosine activating motifs (ITAM), further activating kinases and the protein kinase C pathway, opening Ca^2+^ channels and increasing the intracellular concentration of Ca^2+^. Kinase activation leads to actin remodeling, allowing for phagocytosis and cellular intake of the bound IgG immune complex and activating transcription factors that drive the expression and release of pro-inflammatory cytokines, leading to an antiviral immune response. ADE occurs through the exploitation of FcγR mediated uptake by viral complexes bound by non-neutralizing IgG antibodies, which allows for viral entry and DENV replication in immune cells (119). In neonates born to DENV infected mothers, ADE can occur during a specific window of opportunity. This is when maternally-derived IgG antibodies have decayed to a level where the infant has sub neutralizing levels of antibodies that are still capable of enhancing DENV infection in cells expressing Fc receptors (118).

### 5.2 Antibody-mediated ZIKV control and enhancement mechanisms – a dichotomous relationship

Maternal antibodies serve dual roles in ZIKV pathogenesis, with neutralizing antibodies generated from prior orthoflavivirus infections, particularly DENV, capable of either protective neutralization or pathogenic enhancement through ADE (120). As previously discussed, in ADE, non-neutralizing or sub-neutralizing antibodies facilitate viral cellular entry rather than clearance, increasing infectious severity and raising significant concerns regarding vaccine development strategies that might inadvertently elevate ZIKV vertical transmission risk through cross-reactive humoral responses (121).

Maternal antibodies against ZIKV survive in fetal circulation after crossing the placenta. According to Megli and Coyne (122), some of these antibodies can contribute to immune complexes, exacerbating placental inflammation and injury to the fetus, while others offer passive immunity. To promote transmission, ZIKV has been shown to take advantage of the autophagy pathway by producing exosomes that contain viral proteins and RNA. A different method of viral transmission to the fetus is possible because of the ability of these exosomes to cross the placenta (123). Blocking autophagy has been shown to lower ZIKV transmission, suggesting this mechanism may be important for viral dissemination in pregnancy (124). Apart from inhibiting the interferon response, ZIKV utilizes supplementary immune evasion strategies to improve its survival. Mechanisms include downregulation of antiviral pathways and upregulation of immunological tolerance by viral modulation of placental immunity gene expression (68). This modification may make it possible for ZIKV to survive in the placenta without inducing a strong maternal immune response (125).

### 5.3 Host innate and adaptive immunity towards ZIKV infection

ZIKV reduces the antiviral effects of type I IFN signaling by suppressing the maternal interferon response through the degradation of STAT2 (95). While maternal antibodies can help control the spread of ZIKV, the potential for ADE can worsen the infection (126) (**Table 1**).

**Table 1:** Comparison of immunological factors influencing vertical transmission of DENV and ZIKV.

Factors	DENV	ZIKV
Innate Immunity	Major immune cells involved in the pro-inflammatory phase of pregnancy include dendritic cells, decidual macrophages, and NK cells. Later shifts to anti-inflammatory with Hofbauer cells and Tregs. Hofbauer cells are highly permissive (127).	Trophoblasts generate type III interferons (IFN-λ); Hofbauer cells act as reservoirs for viral loads. Endothelial cells and placental fibroblast cells are also susceptible (68).
Cytokine Response	Decidual macrophages secrete TNF-α and IL-6 in a pro-inflammatory environment, while producing IL-10 in the anti-inflammatory phase (128).	Pro-inflammatory cytokines (TNF-α, IL-6) disrupt placental function (68).
Maternal Antibodies	Maternal neutralizing IgG antibodies protects infants, but declining levels can lead to neonatal death from DENV (52). Non-neutralizing antibodies drive antibody-dependent enhancement (ADE), allowing viral entry into leukocytes via FcγR (129). Sialylation depends on inflammation, while afucosylation increases inflammation and viral uptake, worsening DENV (106).	Maternal neutralizing antibodies may protect, but cross-reactive/non-neutralizing antibodies enhance ZIKV infection (ADE). Antibodies may also form immune complexes in fetus (126).
Immune Evasion Mechanisms	FcyR-mediated pathway to silently enter and replicate in immune cells. Hofbauer and cord blood cells serve as a passage allowing DENV to bypass the immune system (52).	Autophagy and exosome-mediated pathways for viral spread across placenta. Antiviral pathways are downregulated, and immunological tolerance genes are upregulated in the virus’s modulation of placental immunity gene expression (130). Suppresses type I IFN via STAT2 degradation (95).
Key Outcomes/Risks	Infant DENV due to decrease in maternal antibodies; vertical transmission through cord blood stem cells (52).	Congenital ZIKV Syndrome (CZS), microcephaly, neurological damage, impaired neurodevelopment (68).

Recent studies have identified IL-27 as an important host innate immune factor against viral infection in the placenta. Trophoblast organoids express both IL-27 and IL27 receptor constitutively, which can restrict ZIKV infection in the placenta (131). Additionally, decidual natural killer cells act as the front line of defense at the MFI and have shown to kill ZIKV-infected trophoblasts (132). However, the virus also has the capability of escaping detection by NK cells by upregulating MHC class 1 proteins, inhibiting NK cell receptors, demonstrating the ability of the virus to evade innate immunity (133). As previously discussed, ZIKV exacerbates placental damage by inducing a strong inflammatory response at the MFI. High concentrations of pro-inflammatory cytokines, such as IL-6, IL-1β, and TNF-α, are produced by infected trophoblasts and immune cells in the placenta (134). The body’s physiologic antiviral response includes these protective inflammatory mediators. Studies on STAT2-deficient mice, showed heightened vulnerability to ZIKV infection and provide additional evidence of the significance of STAT2 in ZIKV defense (29). Type III interferon (IFN-λ1), which offers paracrine and autocrine protection against viral propagation, has been demonstrated in term placentae to exhibit resistance to ZIKV infection constitutively (15). Duramycin administration, which reduces ZIKV infection rates in both primary placental cells and tissue explant cultures, may be a potential therapeutic approach for limiting viral placental invasion and fetal transmission (16).

The placenta contains specific micro RNAs (miRNAs) that act as genetic silencers by binding the 3’ untranslated region (UTR) of mRNA (135). A selection of these placenta-specific miRNA come from the chromosome 19 miRNA cluster (C19MC). It was recently discovered that this gene cluster protects trophoblast cells by hindering the excessive activation of innate immunity. So, C19MC may not only be important in maintaining immunity at the MFI, but also in protecting the placenta against potentially harmful autoimmunity (136). During ZIKV infection, C19MC attenuates the pathogenesis of the virus limiting its ability to infect and reproduce in the placenta. However, the exact mechanism by which this gene cluster acts is still unknown, as it doesn’t influence the IFN-III pathway or synergize with IFN-λ1 (137).

## 6 Concurrent pathogen infection during gestation

Coinfection represents the simultaneous establishment of multiple infectious agents within a single host organism. This phenomenon presents diagnostic and therapeutic challenges due to overlapping clinical manifestations and potential inter-pathogen interactions that may facilitate enhanced viral replication kinetics (138). This discussion examines gestational complications arising from concurrent infection with DENV or ZIKV alongside other viral pathogens, as well as dual DENV-ZIKV infection. DENV and ZIKV constitute phylogenetically related arboviruses that demonstrate geographic co-circulation in endemic regions (139). Although documented cases of dual infection exist, these pathogens exhibit competitive viral interference, whereby one agent predominates and suppresses replication of the competing virus (138). In areas with established co-circulation of both orthoflaviviruses, concurrent DENV-ZIKV infection in pregnancy is a significant clinical concern.

These viruses share antigenic cross-reactivity and seasonal transmission cycles, creating complex diagnostic challenges. Clinical presentations vary, with some cases demonstrating predominant ZIKV symptomatology (139) while others exhibit more pronounced DENV manifestations (140). DENV-ZIKV dual infections typically lack demonstrable viral synergism (140). The gestational impact of coinfection remains inadequately characterized. Recent data suggests pre-existing dengue immunity can modulate ZIKV pathogenesis during pregnancy. A murine study by Rathore et al. (126) demonstrated that pre-existing DENV antibodies may enhance ZIKV replication through ADE, resulting in elevated viral loads within placental and fetal tissue. Consequent outcomes included increased fetal mortality, enhanced inflammatory responses, and exacerbated placental pathology. These findings corroborate clinical observations suggesting that dual viral exposure may correlate with more severe gestational complications.

In humans, differentiation between the clinical manifestations of co- versus mono-infection with DENV and ZIKV in pregnancy may not be possible (141, 142). Congenital Zika syndrome (CZS) is known to result from isolated ZIKV infection, although co-infection may also be associated with microcephaly, intrauterine growth restriction (IUGR), premature birth, neurological, and neurodevelopmental aftereffects. Because of their structural similarities, especially in envelope proteins, ZIKV and DENV may cause cross-reactivity in serological tests complicating diagnostic testing in pregnancy. During the acute stage of illness, RT-PCR is still the gold standard for detecting viral RNA; however, once viremia goes away, the usefulness of this test decreases.

A study by João et al. (143) examined outcomes in pregnant women co-infected with HIV and ZIKV. The investigators noted that co-infection increased fetal risks. Additional studies have been conducted on coinfection between these arboviruses, including one on a cohort of pregnant women in the south of Mexico (142) and a case report from Colombia (142). These studies did not find that infection with multiple viruses enhanced or synergized clinical outcomes during coinfection. In a recent study Nolan et al. (144) investigated seroprevalence rates of co-infection among laboring mothers in El Salvador and documented elevated antibody titers against DENV and ZIKV. The need for more sophisticated and virus-specific diagnostic algorithms is suggested by the difficulty in attributing infant outcomes to a single pathogen due to the overlap in maternal immune responses (145) Strong maternal screening, molecular diagnostics, and integrated arbovirus surveillance are therefore urgently needed especially in areas endemic to both viruses.

## 7 Future directions

The comparative placental transmission mechanisms of DENV and ZIKV require future multidisciplinary research that connects immunology, virology, and maternal-fetal medicine. ZIKV can directly infect cytotrophoblasts and Hofbauer cells, while DENV infection relies on immune-mediated disruption of placental integrity (64). Most severe cases of dengue may occur because of ADE during secondary infection (146). However, a recent study that examined 619 DENV-infected infants in India questioned this dogma. In the study, primary and secondary DENV infection was determined by finding the ratio of IgM to IgG antibodies using ELISA. During secondary infection, severe cases were associated with the presence of maternal IgG resulting in ADE, while primary infections were associated with higher concentrations of IgM antibodies. Of the 619 total cases, primary infection was noted in 344 infants and children. Of those 344, 202 were severe, and 112 of those severe cases were due to primary infection. There were also 7 fatalities, and 5 of those 7 involved primary infection. The cohort of individuals analyzed were between newborn to 16 years of age, and 60% of children younger than 1 year old. All were positive for IgM, indicating primary infection. The results from this study show that primary infections can cause severe disease in pediatric subjects (146). Such studies open new avenues for improved DENV management in infants and children. However, it is important to note the lack of direct studies on the MFI during DENV infection as this field lacks both *in-vitro* models and true clinical or *in-vivo* studies. Given the dangers that DENV and ZIKV infection present to fetuses and infants, this area of study is crucial for the health of the mother-infant dyad when exposed to arboviral infection during pregnancy (13, 147, 148). Studies highlighting recent outbreaks of arboviral infections demonstrate differences between infections. One study explored an outbreak of ZIKV, DENV, and CHKV in Brazil during 2014-2016. While this outbreak resulted in increased cases of microcephaly, this association was documented with ZIKV and CHKV, not DENV (149). Another examined an increase of microcephaly in Brazil in 2015. This ZIKV outbreak was correlated with more children being born with microcephaly, but previous DENV outbreaks did not have the same association, leading to the conclusion that microcephaly as a result of CZS is unique to ZIKV (150).

The use of placental organoids, single-cell transcriptomics, and *in vivo* imaging, are recommended to identify differential pathways in real time, as well as in situations when there is co-infection or previous exposure to orthoflavivirus. The role of maternal immunity, particularly ADE, where non-neutralizing antibodies, especially from previous DENV exposure, can exacerbate ZIKV infection and result in worse placental and fetal outcomes, requires further investigation (63, 64). Vaccine design is directly affected by the interaction between antibodies and antigens. Due diligence must be taken in the development of vaccines against both viruses to prevent cross-reactive antibodies from exacerbating ADE, especially in pregnant or childbearing women. Despite being in early-phase trials, the current live-attenuated and DNA-based ZIKV vaccines are not yet authorized for general maternal use because of safety concerns regarding fetal exposure and the potential of ADE. The use of DENV vaccinations, such Dengvaxia, has also been restricted in some areas because of ADE-related effects in DENV-naïve people (65). Future research must therefore focus on developing vaccinations (151, 152) that elicit strong, widespread, and non-enhancing neutralizing antibody responses while also incorporating placental safety assessments in preclinical research. To lessen the effects of both viruses on perinatal outcomes, a comprehensive public health response incorporating vaccinations, diagnostics, surveillance, and treatments specific to the pregnancy will be necessary as the climate changes and arbovirus range spreads.

## 8 Conclusion

Both DENV and ZIK demonstrate maternal-fetal transmission capability during gestation, potentially resulting in severe morbidity for both. Elucidating the mechanistic pathways of transplacental viral dissemination is fundamental to developing comprehensive maternal-fetal protective strategies. While these orthoflaviviruses share common immunopathological features and epidemiological characteristics necessitating coordinated diagnostic and prophylactic approaches, their distinct placental invasion mechanisms and pathogenic impacts require pathogen-specific interventions. Future research priorities should emphasize placental barrier integrity maintenance, maternal-fetal immune response modulation, and vaccine safety profiles during pregnancy. Only through such targeted investigative efforts can we effectively mitigate the long-term sequelae associated with congenital orthoflavivirus infections. The compiled evidence presented in this comprehensive review reinforces the critical importance of early detection and therapeutic intervention, which has been demonstrated as the most efficacious strategy for limiting severe maternal and pediatric outcomes.

## References

[1] Arora N Sadovsky Y Dermody TS Coyne CB . Microbial vertical transmission during human pregnancy. Cell Host Microbe. (2017) 21:561–7. doi: 10.1016/j.chom.2017.04.007, PMID: 28494237 PMC6148370

[2] Moffett A Chazara O Colucci F . Maternal allo-recognition of the fetus. Fertil Steril. (2017) 107:1269–72. doi: 10.1016/j.fertnstert.2017.05.001, PMID: 28577615

[3] Mor G Cardenas I Abrahams V Guller S . Inflammation and pregnancy: The role of the immune system at the implantation site. Ann N Y Acad Sci. (2011) 1221:80–7. doi: 10.1111/j.1749-6632.2010.05938.x, PMID: 21401634 PMC3078586

[4] Whitelaw PF Croy BA . CURRENT TOPIC granulated lymphocytes of pregnancy. Placenta (1996) 17:533–43. doi: 10.1016/S0143-4004(96)80070-1, PMID: 8916201

[5] Manaster I Mandelboim O . The unique properties of uterine NK cells. Am J Reprod Immunol. (2010) 63:434–44. doi: 10.1111/j.1600-0897.2009.00794.x, PMID: 20055791

[6] Williams PJ Searle RF Robson SC Innes BA Bulmer JN . Decidual leucocyte populations in early to late gestation normal human pregnancy. J Reprod Immunol. (2009) 82:24–31. doi: 10.1016/j.jri.2009.08.001, PMID: 19732959

[7] Parker EL Silverstein RB Verma S Mysorekar IU . Viral-immune cell interactions at the maternal-fetal interface in human pregnancy. Front Immunol. (2020) 11:522047. doi: 10.3389/fimmu.2020.522047, PMID: 33117336 PMC7576479

[8] Pereira L . Congenital viral infection: traversing the uterine-placental interface. Annu. Rev. Virol. (2025) 5:273–99:. doi: 10.1146/annurev-virology, PMID: 30048217

[9] Samung Y Pengon J Pethrak C Pakparnich P Thaiudomsup S Suksirisawat K . Comprehensive intra-host infection kinetics reveals high arbo-orthoflavivirus transmission potential by neglected vector species, Aedes scutellaris. PLoS Negl Trop Dis. (2025) 19:e0012530. doi: 10.1371/journal.pntd.0012530, PMID: 40327673 PMC12080922

[10] Rakotonirina A Dauga C Pol M Hide M Vuth L Ballan V . Speciation patterns of Aedes mosquitoes in the Scutellaris Group: a mitochondrial perspective. Sci Rep. (2024) 14:10930. doi: 10.1038/s41598-024-61573-7, PMID: 38740928 PMC11091128

[11] Calvez E Pocquet N Malau A Kilama S Taugamoa A Labrousse D . Assessing entomological risk factors for arboviral disease transmission in the French Territory of the Wallis and Futuna Islands. PLoS Negl Trop Dis. (2020) 14:e0008250. doi: 10.1371/journal.pntd.0008250, PMID: 32401756 PMC7219742

[12] Modahl CM Chowdhury A Low DHW Manuel MC Missé D Kini RM . Midgut transcriptomic responses to dengue and chikungunya viruses in the vectors Aedes albopictus and Aedes malayensis. Sci Rep. (2023) 13:11271. doi: 10.1038/s41598-023-38354-9, PMID: 37438463 PMC10338677

[13] Mlakar J Korva M Tul N Popović M Poljšak-Prijatelj M Mraz J . Zika virus associated with microcephaly. New Engl J Med. (2016) 374:951–8. doi: 10.1056/nejmoa1600651, PMID: 26862926

[14] Miner JJ Diamond MS . Zika virus pathogenesis and tissue tropism. Cell Host Microbe. (2017) 21:134–42. doi: 10.1016/j.chom.2017.01.004, PMID: 28182948 PMC5328190

[15] Bayer A Lennemann NJ Ouyang Y Bramley JC Morosky S Marques ETDA . Type III interferons produced by human placental trophoblasts confer protection against zika virus infection. Cell Host Microbe. (2016) 19:705–12. doi: 10.1016/j.chom.2016.03.008, PMID: 27066743 PMC4866896

[16] Quicke KM Bowen JR Johnson EL McDonald CE Ma H O’Neal JT . Zika virus infects human placental macrophages. Cell Host Microbe. (2016) 20:83–90. doi: 10.1016/j.chom.2016.05.015, PMID: 27247001 PMC5166429

[17] Watanabe S Vasudevan SG . Clinical and experimental evidence for transplacental vertical transmission of flaviviruses. Antiviral Res. (2023) 210:105512. doi: 10.1016/j.antiviral.2022.105512, PMID: 36572192

[18] Nogueira R Muniz A Coelho J Brasil P Lopes V Ribeiro C . Perinatal Transmission of Dengue: A Report of 7 Cases. J. Pediatr. (2013) 163:1514–6. doi: 10.1016/j.jpeds.2013.06.040, PMID: 23916226

[19] Honein MA Dawson AL Petersen EE Jones AM Lee EH Yazdy MM . Birth defects among fetuses and infants of US women with evidence of possible zika virus infection during pregnancy. JAMA - J Am Med Assoc. (2017) 317:59–68. doi: 10.1001/jama.2016.19006, PMID: 27960197

[20] Brasil P Pereira JP Moreira ME Ribeiro Nogueira RM Damasceno L Wakimoto M . Zika virus infection in pregnant women in rio de janeiro. New Engl J Med. (2016) 375:2321–34. doi: 10.1056/nejmoa1602412, PMID: 26943629 PMC5323261

[21] Rabelo K de Souza Campos Fernandes RC Souza LJ de Louvain de Souza T Santos FBd Guerra Nunes PC . Placental histopathology and clinical presentation of severe congenital zika syndrome in a human immunodeficiency virus-exposed uninfected infant. Front Immunol. (2017) 8:1704. doi: 10.3389/fimmu.2017.01704, PMID: 29270171 PMC5725436

[22] de Noronha L Zanluca C Burger M Suzukawa AA Azevedo M Rebutini PZ . Zika virus infection at different pregnancy stages: anatomopathological findings, target cells and viral persistence in placental tissues. Front Microbiol. (2018) 9:2266. doi: 10.3389/fmicb.2018.02266, PMID: 30337910 PMC6180237

[23] Noronha L Zanluca C Azevedo MLV Luz KG Santos CNDd . Zika virus damages the human placental barrier and presents marked fetal neurotropism. Mem Inst Oswaldo Cruz. (2016) 111:287–93. doi: 10.1590/0074-02760160085, PMID: 27143490 PMC4878297

[24] Paixão ES Teixeira MG Costa M daCN Rodrigues LC . Dengue during pregnancy and adverse fetal outcomes: A systematic review and meta-analysis. Lancet Infect Dis. (2016) 16:857–65. doi: 10.1016/S1473-3099(16)00088-8, PMID: 26949028

[25] Basurko C Carles G Youssef M Guindi WEL . Maternal and foetal consequences of dengue fever during pregnancy. Eur J Obstet Gynecology Reprod Biol. (2009) 147:29–32. doi: 10.1016/j.ejogrb.2009.06.028, PMID: 19632027

[26] Nunes PCG Paes MV de Oliveira CAB Soares ACG de Filippis AMB Lima M da RQ . Detection of dengue NS1 and NS3 proteins in placenta and umbilical cord in fetal and maternal death. J Med Virol. (2016) 88:1448–52. doi: 10.1002/jmv.24479, PMID: 26792253

[27] Dengue on the rise: get the facts. Atlanta, Georgia: CDC (2024). Available online at: https://www.cdc.gov/dengue/stories/dengue-on-the-rise-get-the-facts.html.

[28] Dejnirattisai W Supasa P Wongwiwat W Rouvinski A Barba-Spaeth G Duangchinda T . Dengue virus sero-cross-reactivity drives antibody-dependent enhancement of infection with zika virus. Nat Immunol. (2016) 17:1102–8. doi: 10.1038/ni.3515, PMID: 27339099 PMC4994874

[29] Bardina SV Bunduc P Tripathi S Duehr J Frere JJ Brown JA . Enhancement of Zika virus pathogenesis by preexisting antiflavivirus immunity. Science. (2017) 356:175–80. doi: 10.1126/science.aal4365, PMID: 28360135 PMC5714274

[30] Zimmerman MG Quicke KM O’Neal JT Arora N Machiah D Priyamvada L . Cross-reactive dengue virus antibodies augment zika virus infection of human placental macrophages. Cell Host Microbe. (2018) 24:731–742.e6. doi: 10.1016/j.chom.2018.10.008, PMID: 30439342 PMC6394860

[31] Liao KC Xie X Sundstrom AKB Lim XN Tan KK Zhang Y . Dengue and Zika RNA-RNA interactomes reveal pro- and anti-viral RNA in human cells. Genome Biol. (2023) 24. doi: 10.1186/s13059-023-03110-9, PMID: 38053173 PMC10696742

[32] Messina JP Brady OJ Scott TW Zou C Pigott DM Duda KA . Global spread of dengue virus types: mapping the 70 year history. Trends Microbiol. (2014) 22:138–46. doi: 10.1016/j.tim.2013.12.011, PMID: 24468533 PMC3946041

[33] Areas with risk of dengue. Atlanta, Georgia: CDC (2025). Available online at: https://www.cdc.gov/dengue/areas-with-risk/index.html.

[34] Countries & Territories at Risk for Zika. Atlanta, Georgia: CDC (2025). Available online at: https://www.cdc.gov/zika/geo/index.html.

[35] Gubler DJ Vasilakis N Musso D . History and emergence of zika virus. J Infect Dis. (2017) 216:S860–7. doi: 10.1093/infdis/jix451, PMID: 29267917 PMC5853376

[36] Rathore SS Oberoi S Hilliard J Raja R Ahmed NK Vishwakarma Y . Maternal and foetal-neonatal outcomes of dengue virus infection during pregnancy. Trop Med Int Health. (2022) 27:619–29. doi: 10.1111/tmi.13783, PMID: 35689528

[37] Ferdous B Rahman MM Halim T Khatun H Mohammad A . Maternal and neonatal outcomes of dengue fever in pregnancy: A cross-sectional study. Med Today. (2025) 37:5–8. doi: 10.3329/medtoday.v37i1.79247

[38] Kularatne SA Dalugama C . Dengue infection: Global importance, immunopathology and management. Clin Med J R Coll Physicians London. (2022) 22:9–13. doi: 10.7861/clinmed.2021-0791, PMID: 35078789 PMC8813012

[39] Duarte G Neto ARB Kreitchmann R Menezes MLB Miranda AEB Travassos AGA . Prevention, diagnosis, and treatment protocol of dengue during pregnancy and the postpartum period. Rev Bras Ginecologia e Obstetricia. (2024) 46. doi: 10.61622/rbgo/2024rbgo73, PMID: 38994458 PMC11239217

[40] Jahan I Alam L Akter S . Dengue in pregnancy: A systematic review and meta-analysis. JMSRP. (2024) 5:41–9. doi: 10.70818/iarjmsr.2024.v05i06.0143

[41] Shabil M Khatib MN Zahiruddin QS Rekha MM Kaur M Rani B . Dengue infection during pregnancy and adverse birth outcomes: A systematic review and meta-analysis. Rev Med Virol. (2024) 34. doi: 10.1002/rmv.2582, PMID: 39245582

[42] Lahon A Arya RP Banerjea AC . Dengue virus dysregulates master transcription factors and PI3K/AKT/mTOR signaling pathway in megakaryocytes. Front Cell Infect Microbiol. (2021) 11:715208. doi: 10.3389/fcimb.2021.715208, PMID: 34513730 PMC8427595

[43] Chong V Tan JZL Arasoo VJT . Dengue in Pregnancy: A Southeast Asian Perspective. Trop Med Infect Dis. (2023) 8:86. doi: 10.3390/tropicalmed8020086, PMID: 36828502 PMC9964792

[44] Cerqueira-Silva T Rodrigues LC Pearce N Teixeira MG Costa M daCN Cardim L . Perinatal outcomes of symptomatic chikungunya, dengue and Zika infection during pregnancy in Brazil: a registry-based cohort study. Nat Commun. (2025) 16:7207. doi: 10.1038/s41467-025-62640-x, PMID: 40764313 PMC12325709

[45] Yin X Zhong X Pan S . Vertical transmission of dengue infection: the first putative case reported in China. Rev Inst Med Trop Sao Paulo. (2016) 58:90. doi: 10.1590/S1678-9946201658090, PMID: 27982356 PMC5147720

[46] Golding MAJ Noble SAA Khouri NK Layne-Yarde RNA Ali I Sandiford SL . Natural vertical transmission of dengue virus in Latin America and the Caribbean: highlighting its detection limitations and potential significance. Parasit Vectors. (2023) 16:442. doi: 10.1186/s13071-023-06043-1, PMID: 38017450 PMC10685567

[47] Arragain L Dupont-Rouzeyrol M O'Connor O Sigur N Grangeon JP Huguon E . Vertical Transmission of Dengue Virus in the Peripartum Period and Viral Kinetics in Newborns and Breast Milk: New Data. J Pediatric Infect Dis Soc. (2017) 6:324–31. doi: 10.1093/jpids/piw058, PMID: 27760799

[48] Ribeiro CF Lopes VGS Brasil P Coelho J Muniz AG Nogueira RMR . Perinatal transmission of dengue: A report of 7 cases. J Pediatr. (2013) 163:1514–6. doi: 10.1016/j.jpeds.2013.06.040, PMID: 23916226

[49] Sinhabahu VP Sathananthan R Malavige GN . Perinatal transmission of dengue: A case report. BMC Res Notes. (2014) 7:700–1. doi: 10.1186/1756-0500-7-795, PMID: 25394748 PMC4237779

[50] Usama S Assawawiroonhakarn S Soonsawad S . Vertical dengue transmission complicated with neonatal encephalitis. BMJ Case Rep. (2023) 16. doi: 10.1136/bcr-2023-256476, PMID: 38160035 PMC10759012

[51] Lee PX Ong LC Libau EA Alonso S . Relative Contribution of Dengue IgG Antibodies Acquired during Gestation or Breastfeeding in Mediating Dengue Disease Enhancement and Protection in Type I Interferon Receptor-Deficient Mice. PLoS Negl Trop Dis. (2016) 10:e0004805. doi: 10.1371/journal.pntd.0004805, PMID: 27341339 PMC4920417

[52] Nguyen TM Huan VT Reda A Morsy S Nam Giang HT Tri VD . Clinical features and outcomes of neonatal dengue at the Children’s Hospital 1, Ho Chi Minh, Vietnam. J Clin Virol. (2021) 138. doi: 10.1016/j.jcv.2021.104758, PMID: 33862538

[53] Arabdin M Khan A Zia S Khan S Khan GS Shahid M . Frequency and severity of thrombocytopenia in neonatal sepsis. Cureus. (2022) 14:e22665. doi: 10.7759/cureus.22665, PMID: 35386168 PMC8967110

[54] Nielsen-Saines K Brasil P Fuller TL . Zika virus. In: Infectious diseases of the fetus and newborn infant. Philadelphia, PA: Elsevier (2025). doi: 10.1016/B978-0-323-79525-8.00051-2

[55] Guardado K López-Balderas N Morales-Romero J Sampieri CL Zenteno-Cuevas R Álvarez-Bañuelos MT . Zika virus infection in asymptomatic pregnant women. Infect Dis Rep. (2025) 17. doi: 10.3390/idr17010002, PMID: 39846705 PMC11755599

[56] Hcini N Lambert V Carod JF Mathieu M Carles G Picone O . Emerging and re-emerging infectious diseases in pregnant women in an amazonian region: a large retrospective study from French Guiana. Eur J Clin Microbiol Infect Dis. (2024) 43:1081–90. doi: 10.1007/s10096-024-04813-y, PMID: 38573394

[57] Wongsawat J Thamthitiwat S Hicks VJ Uttayamakul S Teepruksa P Sawatwong P . Characteristics, risk factors, and outcomes related to Zika virus infection during pregnancy in Northeastern Thailand: A prospective pregnancy cohort study, 2018–2020. PLoS Negl Trop Dis. (2024) 18. doi: 10.1371/journal.pntd.0012176, PMID: 38758964 PMC11139345

[58] Deshpande GR Sapkal GN Salunke A Gunjikar R Tadkalkar N Shinde P . An outbreak of Zika virus in western India in the metropolis of Pune in the monsoon of 2024. J Infect Public Health. (2025) 18. doi: 10.1016/j.jiph.2025.102720, PMID: 40043420

[59] Venancio FA Quilião ME Gabeira SC deO Carvalho AT de Leite SHDS de Lima SMB . Early and long-term adverse outcomes of *in utero* zika exposure. Pediatrics. (2025) 155. doi: 10.1542/peds.2024-067552, PMID: 39814049 PMC11832048

[60] Secord E John McGrath E Chandra Sahoo K Roopchand Sahay M Hedley phy PL . The perinatal health challenges of emerging and re-emerging infectious diseases: A narrative review. Lausanne, Switzerland: Frontiers in Public Health. (2023). 10.3389/fpubh.2022.1039779 PMC9850110 36684933

[61] Ghosh S Salan T Riotti J Ramachandran A Gonzalez IA Bandstra ES . Brain MRI segmentation of Zika-Exposed normocephalic infants shows smaller amygdala volumes. PLoS One. (2023) 18. doi: 10.1371/journal.pone.0289227, PMID: 37506075 PMC10381087

[62] Sher AA Lao YT Coombs KM . HLA-A, HSPA5, IGFBP5 and PSMA2 are restriction factors for zika virus growth in astrocytic cells. Viruses. (2023) 15. doi: 10.3390/v15010097, PMID: 36680137 PMC9863221

[63] Mercado M Ailes EC Daza M Tong VT Osorio J Valencia D . Zika virus detection in amniotic fluid and Zika-associated birth defects. Am. J. Obstet. Gynecol. (2020) 222:610.e1–610.e13. doi: 10.1016/j.ajog.2020.01.009, PMID: 31954155 PMC7477618

[64] Brown JA Singh G Acklin JA Lee S Duehr JE Chokola AN . Dengue virus immunity increases zika virus-induced damage during pregnancy. Immunity. (2019) 50:751–762.e5. doi: 10.1016/j.immuni.2019.01.005, PMID: 30737148 PMC6947917

[65] Shukla R Ramasamy V Shanmugam RK Ahuja R Khanna N . Antibody-dependent enhancement: A challenge for developing a safe dengue vaccine. Front Cell Infect Microbiol. (2020) 10:572681. doi: 10.3389/fcimb.2020.572681, PMID: 33194810 PMC7642463

[66] Parums DV . A review of emerging viral pathogens and current concerns for vertical transmission of infection. Med Sci Monit. (2024) 30:e947335. doi: 10.12659/MSM.947335, PMID: 39578400 PMC11600638

[67] Cao B Diamond MS Mysorekar IU . Maternal-fetal transmission of zika virus: Routes and signals for infection. J Interferon Cytokine Res. (2017) 37:287–94. doi: 10.1089/jir.2017.0011, PMID: 28402153 PMC5512303

[68] Benazzato C Lojudice F Pöehlchen F Leite PEC Manucci AC van der Linden V . Zika virus vertical transmission induces neuroinflammation and synapse impairment in brain cells derived from children born with Congenital Zika Syndrome. Sci Rep. (2024) 14. doi: 10.1038/s41598-024-65392-8, PMID: 39097642 PMC11297915

[69] Rosario-Falero JM Morantes-Betancourt GP Claudio N Pérez R Reyes-Bou Z . Ophthalmologic Findings in a Premature Infant leading to a Zika Diagnosis during the COVID-19 Pandemic: A Case Report. P R Health Sci J. (2024) 43:54–6., PMID: 38512762

[70] Ventura CV Bandstra ES Fernandez MP Cooper JM Saigal GM Bauer CR . First locally acquired congenital zika syndrome case in the United States: Neonatal clinical manifestations. Ophthalmic Surg Lasers Imaging Retina. (2018) 49:e93–8. doi: 10.3928/23258160-20180907-14, PMID: 30222826

[71] Leonhard SE Mandarakas MR Gondim FAA Bateman K Ferreira MLB Cornblath DR . Diagnosis and management of Guillain–Barré syndrome in ten steps. Nat Rev Neurol. (2019) 15:671–83. doi: 10.1038/s41582-019-0250-9, PMID: 31541214 PMC6821638

[72] Bellanti R Rinaldi S . Guillain-Barré syndrome: a comprehensive review. Eur J Neurol. (2024) 31:e16365. doi: 10.1111/ene.16365, PMID: 38813755 PMC11235944

[73] Dirlikov E Medina NA Major CG Munoz-Jordan JL Luciano CA Rivera-Garcia B . Acute zika virus infection as a risk factor for guillain-barré Syndrome in Puerto Rico. JAMA. (2017) 318:1498–500. doi: 10.1001/jama.2017.11483, PMID: 29049645 PMC5817969

[74] Parra B Lizarazo J Jiménez-Arango JA Zea-Vera AF González-Manrique G Vargas J . Guillain–barré Syndrome associated with zika virus infection in Colombia. New Engl J Med. (2016) 375:1513–23. doi: 10.1056/NEJMoa1605564, PMID: 27705091

[75] Lim CS Kaisbain N Lim WJ . A rare combination: dengue fever complicated with guillain-barre syndrome. Cureus. (2023) 15:e40957. doi: 10.7759/cureus.40957, PMID: 37503499 PMC10369010

[76] Garvey M . Neonatal infectious disease: A major contributor to infant mortality requiring advances in point-of-care diagnosis. Antibiotics. (2024) 13. doi: 10.3390/antibiotics13090877, PMID: 39335050 PMC11428345

[77] Ades AE Soriano-Arandes A Alarcon A Bonfante F Thorne C Peckham CS . Vertical transmission of Zika virus and its outcomes: a Bayesian synthesis of prospective studies. Lancet Infect Dis. (2021) 21:537–45. doi: 10.1016/S1473-3099(20)30432-1, PMID: 33068528 PMC7992034

[78] Malavige GN Ogg GS . Molecular mechanisms in the pathogenesis of dengue infections. Trends Mol Med. (2024) 30:484–98. doi: 10.1016/j.molmed.2024.03.006, PMID: 38582622

[79] Teo A Chua CLL Chia PY Yeo TW . Insights into potential causes of vascular hyperpermeability in dengue. PLoS Pathog. (2021) 17. doi: 10.1371/journal.ppat.1010065, PMID: 34882753 PMC8659665

[80] Huang YH Lei HY Liu HS Lin YS Liu CC Yeh TM . Dengue virus infects human endothelial cells and induces IL-6 and IL-8 production. Am J Trop Med Hyg. (2000) 63:71–5. doi: 10.4269/ajtmh.2000.63.71, PMID: 11357999

[81] Meuren LM Prestes EB Papa MP de Carvalho LRP Mustafá YM da Costa LS . Infection of endothelial cells by dengue virus induces ROS production by different sources affecting virus replication, cellular activation, death and vascular permeability. Front Immunol. (2022) 13:810376. doi: 10.3389/fimmu.2022.810376, PMID: 35185902 PMC8847576

[82] Kanakalatha D Radha S Nambisan B . Maternal and fetal outcome of dengue fever during pregnancy. Int J Reprod Contracept Obstet Gynecol. (2016) 5:3959–64. doi: 10.18203/2320-1770.ijrcog20163871, PMID: 22297282

[83] Naik S Robinson ML Alexander M Chandanwale A Sambarey P Kinikar A . Intensified short symptom screening program for dengue infection during pregnancy, India. Emerg Infect Dis. (2020) 26:738–43. doi: 10.3201/eid2604.191476, PMID: 32186485 PMC7101120

[84] Vats A Ho TC Puc I Chen YJ Chang CH Chien YW . Evidence that hematopoietic stem cells in human umbilical cord blood is infectable by dengue virus: proposing a vertical transmission candidate. Heliyon. (2021) 7. doi: 10.1016/j.heliyon.2021.e06785, PMID: 33981874 PMC8082560

[85] Watanabe S Chan KWK Tan NWW Mahid MBA Chowdhury A Chang KTE . Experimental evidence for a high rate of maternal-fetal transmission of dengue virus in the presence of antibodies in immunocompromised mice. EBioMedicine. (2022) 77:103930. doi: 10.1016/j.ebiom.2022.103930, PMID: 35290828 PMC8921544

[86] Ribeiro CF Lopes VGS Brasil P Pires ARC Rohloff R Nogueira RMR . Dengue infection in pregnancy and its impact on the placenta. Int J Infect Dis. (2017) 55:109–12. doi: 10.1016/j.ijid.2017.01.002, PMID: 28088588

[87] Zhang Y Sheng Z Chen Q Zhou A Cao J Xue F . Neutrophil infiltration leads to fetal growth restriction by impairing the placental vasculature in DENV-infected pregnant mice. EBioMedicine. (2023) 95:104739. doi: 10.1016/j.ebiom.2023.104739, PMID: 37544202 PMC10432184

[88] Ahuja S Muntode Gharde P . A narrative review of maternal and perinatal outcomes of dengue in pregnancy. Cureus. (2023) 15. doi: 10.7759/cureus.48640, PMID: 38090413 PMC10711354

[89] Radan A-P Renz P Raio L Villiger A-S Haesler V Trippel M . SARS-CoV-2 replicates in the placenta after maternal infection during pregnancy. Front Med (Lausanne). (2024) 11:1439181. doi: 10.3389/fmed.2024.1439181, PMID: 39296889 PMC11409086

[90] Hecht JL Quade B Deshpande V Mino-Kenudson M Ting DT Desai N . SARS-CoV-2 can infect the placenta and is not associated with specific placental histopathology: a series of 19 placentas from COVID-19-positive mothers. Modern Pathol. (2020) 33:2092–103. doi: 10.1038/s41379-020-0639-4, PMID: 32741970 PMC7395938

[91] Chen J Neil JA Tan JP Rudraraju R Mohenska M Sun YBY . A placental model of SARS-CoV-2 infection reveals ACE2-dependent susceptibility and differentiation impairment in syncytiotrophoblasts. Nat Cell Biol. (2023) 25:1223–34. doi: 10.1038/s41556-023-01182-0, PMID: 37443288 PMC10415184

[92] Dubucs C Dupuis N Jourdes A Delobel P Auzet C Van Acker N . Placental lesions induced by Omicron SARS-CoV-2 in a pregnant woman treated with anti-CD20. Placenta. (2025) 165:1–3. doi: 10.1016/j.placenta.2025.03.015, PMID: 40153925

[93] Suresh S Britt JL Freedman A Keenan-Devlin L Ernst LM Lewandowski W . Association between SARS-CoV-2 infection during pregnancy and placental pathology assessed by histology and gene expression. Pregnancy. (2025) 1. doi: 10.1002/pmf2.70052 PMC13344563 42596967

[94] Movahed F Haji Hosseini F Heidari A Dehbozorgi M Ataei M Vahidi F . COVID-19 vertical transmission from mothers to neonates: A systematic review and meta-analysis of 204 studies. J Infect Public Health. (2025) 18:102825. doi: 10.1016/j.jiph.2025.102825, PMID: 40466390

[95] Grant A Ponia SS Tripathi S Balasubramaniam V Miorin L Sourisseau M . Zika virus targets human STAT2 to inhibit type i interferon signaling. Cell Host Microbe. (2016) 19:882–90. doi: 10.1016/j.chom.2016.05.009, PMID: 27212660 PMC4900918

[96] Ren W Fu C Zhang Y Ju X Jiang X Song J . Zika virus NS5 protein inhibits type I interferon signaling via CRL3 E3 ubiquitin ligase-mediated degradation of STAT2. Proc Natl Acad Sci. (2024) 121. doi: 10.1073/pnas.2403235121, PMID: 39145933 PMC11348293

[97] Kung P-L Chou T-W Lindman M Chang NP Estevez I Buckley BD . Zika virus-induced TNF-α signaling dysregulates expression of neurologic genes associated with psychiatric disorders. J Neuroinflamm. (2022) 19:100. doi: 10.1186/s12974-022-02460-8, PMID: 35462541 PMC9036774

[98] Warrington JP Drummond HA Granger JP Ryan MJ . Placental ischemia-induced increases in brain water content and cerebrovascular permeability: role of TNF-α. Am J Physiol Regul Integr Comp Physiol. (2015) 309:R1425–31. doi: 10.1152/ajpregu.00372.2015, PMID: 26400187 PMC4698405

[99] Goldman-Wohl D Yagel S . United we stand not dividing: The syncytiotrophoblast and cell senescence. Placenta. (2014) 35:341–4. doi: 10.1016/j.placenta.2014.03.012, PMID: 24709558

[100] Hermanns K Göhner C Kopp A Schmidt A Merz WM Markert UR . Zika virus infection in human placental tissue explants is enhanced in the presence of dengue virus antibodies *in-vitro*. Emerg Microbes Infect. (2018) 7:198. doi: 10.1038/s41426-018-0199-6, PMID: 30504926 PMC6274641

[101] Miranda J Martín-Tapia D Valdespino-Vázquez Y Alarcón L Espejel-Nuñez A Guzmán-Huerta M . Syncytiotrophoblast of placentae from women with zika virus infection has altered tight junction protein expression and increased paracellular permeability. Cells. (2019) 8. doi: 10.3390/cells8101174, PMID: 31569528 PMC6829373

[102] Wu H Huang X-Y Sun M-X Wang Y Zhou H-Y Tian Y . Zika virus targets human trophoblast stem cells and prevents syncytialization in placental trophoblast organoids. Nat Commun. (2023) 14:5541. doi: 10.1038/s41467-023-41158-0, PMID: 37684223 PMC10491779

[103] Nowakowski TJ Pollen AA Di Lullo E Sandoval-Espinosa C Bershteyn M Kriegstein AR . Expression Analysis Highlights AXL as a Candidate Zika Virus Entry Receptor in Neural Stem Cells. Cell Stem Cell. (2016) 18:591–6. doi: 10.1016/j.stem.2016.03.012, PMID: 27038591 PMC4860115

[104] Oeyen M Heymann CJF Jacquemyn M Daelemans D Schols D . The role of TIM-1 and CD300a in zika virus infection investigated with cell-based electrical impedance. Biosens (Basel). (2024) 14. doi: 10.3390/bios14080362, PMID: 39194591 PMC11352571

[105] Hastings AK Yockey LJ Jagger BW Hwang J Uraki R Gaitsch HF . TAM receptors are not required for zika virus infection in mice. Cell Rep. (2017) 19:558–68. doi: 10.1016/j.celrep.2017.03.058, PMID: 28423319 PMC5485843

[106] Muthuraj PG Sahoo PK Kraus M Bruett T Annamalai AS Pattnaik A . Zika virus infection induces endoplasmic reticulum stress and apoptosis in placental trophoblasts. Cell Death Discov. (2021) 7. doi: 10.1038/s41420-020-00379-8, PMID: 33500388 PMC7838309

[107] Weisblum Y Oiknine-Djian E Vorontsov OM Haimov-Kochman R Zakay-Rones Z Meir K . Zika virus infects early- and midgestation human maternal decidual tissues, inducing distinct innate tissue responses in the maternal-fetal interface. J Virol. (2017) 91. doi: 10.1128/jvi.01905-16, PMID: 27974560 PMC5286880

[108] Turpin J El-Safadi D Lebeau G Frumence E Desprès P Viranaïcken W . Chop pro-apoptotic transcriptional program in response to er stress is hacked by zika virus. Int J Mol Sci. (2021) 22. doi: 10.3390/ijms22073750, PMID: 33916874 PMC8038490

[109] Mohd Ropidi MI Khazali AS Nor Rashid N Yusof R . Endoplasmic reticulum: A focal point of Zika virus infection. J BioMed Sci. (2020) 27. doi: 10.1186/s12929-020-0618-6, PMID: 31959174 PMC6971992

[110] Gladwyn-Ng I Cordón-Barris L Alfano C Creppe C Couderc T Morelli G . Stress-induced unfolded protein response contributes to Zika virus-associated microcephaly. Nat Neurosci. (2018) 21:63–73. doi: 10.1038/s41593-017-0038-4, PMID: 29230053

[111] Tan Z Zhang W Sun J Fu Z Ke X Zheng C . ZIKV infection activates the IRE1-XBP1 and ATF6 pathways of unfolded protein response in neural cells. J Neuroinflamm. (2018) 15. doi: 10.1186/s12974-018-1311-5, PMID: 30241539 PMC6151056

[112] Turpin J El Safadi D Lebeau G Krejbich M Chatelain C Desprès P . Apoptosis during ZIKA virus infection: too soon or too late? Int J Mol Sci. (2022) 23. doi: 10.3390/ijms23031287, PMID: 35163212 PMC8835863

[113] Fernandes-Santos C Azeredo EL de . Innate immune response to dengue virus: toll-like receptors and antiviral response. Viruses. (2022) 14. doi: 10.3390/v14050992, PMID: 35632732 PMC9147118

[114] Luo H Winkelmann ER Fernandez-Salas I Li L Mayer SV Danis-Lozano R . Zika, dengue and yellow fever viruses induce differential anti-viral immune responses in human monocytic and first trimester trophoblast cells. Antiviral Res. (2018) 151:55–62. doi: 10.1016/j.antiviral.2018.01.003, PMID: 29331320 PMC5844857

[115] Castanha PMS Braga C Cordeiro MT Souza AI Silva CD Martelli CMT . Placental transfer of dengue virus (DENV)-specific antibodies and kinetics of DENV infection-enhancing activity in Brazilian infants. J Infect Dis. (2016) 214:265–72. doi: 10.1093/infdis/jiw143, PMID: 27056951 PMC4918828

[116] O’Driscoll M Buddhari D Huang AT Waickman A Kaewhirun S Iamsirithaworn S . Maternally derived antibody titer dynamics and risk of hospitalized infant dengue disease. Proc Natl Acad Sci. (2023) 120. doi: 10.1073/pnas.2308221120, PMID: 37774093 PMC10576102

[117] Thulin NK Brewer RC Sherwood R Bournazos S Edwards KG Ramadoss NS . Maternal anti-dengue igG fucosylation predicts susceptibility to dengue disease in infants. Cell Rep. (2020) 31. doi: 10.1016/j.celrep.2020.107642, PMID: 32402275 PMC7344335

[118] Simmons CP Chau TNB Tran TT Nguyen MT Dang MH Nguyen TT . Maternal antibody and viral factors in the pathogenesis of dengue virus in infants. J Infect Dis. (2007) 196:416–24. doi: 10.1086/519170, PMID: 17597456 PMC4333207

[119] Bournazos S Gupta A Ravetch JV . The role of IgG Fc receptors in antibody-dependent enhancement. Nat Rev Immunol. (2020) 20:633–43. doi: 10.1038/s41577-020-00410-0, PMID: 32782358 PMC7418887

[120] Sawant J Patil A Kurle S . A review: understanding molecular mechanisms of antibody-dependent enhancement in viral infections. Vaccines (Basel). (2023) 11. doi: 10.3390/vaccines11071240, PMID: 37515055 PMC10384352

[121] Thomas S Smatti MK Ouhtit A Cyprian FS Almaslamani MA Thani A . Antibody-Dependent Enhancement (ADE) and the role of complement system in disease pathogenesis. Mol Immunol. (2022) 152:172–82. doi: 10.1016/j.molimm.2022.11.010, PMID: 36371813 PMC9647202

[122] Megli CJ Coyne CB . Infections at the maternal–fetal interface: an overview of pathogenesis and defence. Nat Rev Microbiol. (2022) 20:67–82. doi: 10.1038/s41579-021-00610-y, PMID: 34433930 PMC8386341

[123] Zhou W Woodson M Sherman MB Neelakanta G Sultana H . Exosomes mediate Zika virus transmission through SMPD3 neutral Sphingomyelinase in cortical neurons. Emerg Microbes Infect. (2019) 8:307–26. doi: 10.1080/22221751.2019.1578188, PMID: 30866785 PMC6455149

[124] Cao B Parnell LA Diamond MS Mysorekar IU . Inhibition of autophagy limits vertical transmission of Zika virus in pregnant mice. J Exp Med. (2017) 214:2303–13. doi: 10.1084/jem.20170957, PMID: 28694387 PMC5551583

[125] Bhatnagar J Rabeneck DB Martines RB Reagan-Steiner S Ermias Y Estetter LBC . Zika virus RNA replication and persistence in brain and placental tissue. Emerg Infect Dis. (2017) 23:405–14. doi: 10.3201/eid2303.161499, PMID: 27959260 PMC5382738

[126] St. John AL Rathore APS . Adaptive immune responses to primary and secondary dengue virus infections. Nat Rev Immunol. (2019) 19:218–30. doi: 10.1038/s41577-019-0123-x, PMID: 30679808

[127] Reyes L Golos TG . Hofbauer cells: Their role in healthy and complicated pregnancy. Front Immunol. (2018) 9:2628. doi: 10.3389/fimmu.2018.02628, PMID: 30498493 PMC6249321

[128] Villalobos-Sánchez E Burciaga-Flores M Zapata-Cuellar L Camacho-Villegas TA Elizondo-Quiroga DE . Possible routes for zika virus vertical transmission in human placenta: A comprehensive review. Viral Immunol. (2022) 35:392–403. doi: 10.1089/vim.2021.0199, PMID: 35506896

[129] De Aguiar GPCG Da Silva Leite CMG Dias B Vasconcelos SMM De Moraes RA De Moraes MEA . Evidence for host epigenetic signatures arising from arbovirus infections: A systematic review. Front Immunol. (2019) 10:1207. doi: 10.3389/fimmu.2019.01207, PMID: 31214179 PMC6554415

[130] Zhang S Chu WC Lai RC Lim SK Hui JHP Toh WS . Exosomes derived from human embryonic mesenchymal stem cells promote osteochondral regeneration. Osteoarthritis Cartilage. (2016) 24:2135–40. doi: 10.1016/j.joca.2016.06.022, PMID: 27390028

[131] Merlino MS Barksdale B Negatu SG Philip DT Clements RL Miller-Ensminger T . Interleukin-27 is antiviral against Zika virus at the maternal-fetal interface. Nat Commun. (2025) 17:652. doi: 10.1038/s41467-025-67378-0, PMID: 41413389 PMC12816022

[132] Sen Santara S Crespo ÂC Mulik S Ovies C Boulenouar S Strominger JL . Decidual NK cells kill Zika virus–infected trophoblasts. Proc Natl Acad Sci. (2021) 118. doi: 10.1073/pnas.2115410118, PMID: 34785597 PMC8617421

[133] Glasner A Oiknine-Djian E Weisblum Y Diab M Panet A Wolf DG . Zika virus escapes NK cell detection by upregulating major histocompatibility complex class I molecules. J Virol. (2017) 91. doi: 10.1128/JVI.00785-17, PMID: 28878071 PMC5660495

[134] Rabelo K de Souza LJ Salomão NG MaChado LN Pereira PG Portari EA . Zika induces human placental damage and inflammation. Front Immunol. (2020) 11:2146. doi: 10.3389/fimmu.2020.02146, PMID: 32983175 PMC7490298

[135] Aagaard K Seferovic M Hamilton M Hu M Gorchakov R Murray K . 22: Identifying zika virus (ZIKV)-silenced gene targets in the placenta at a transcriptome-wide scale using AGO-clip sequencing technology. Am J Obstet Gynecol. (2018) 218:S18–9. doi: 10.1016/j.ajog.2017.10.433

[136] Mouillet J-F Ouyang Y Sadovsky E Kothandan VK Sorenson HL Badeau LJ . The Chromosome 19 miRNA cluster guards trophoblasts against overacting innate immunity. Commun Biol. (2025) 8:1511. doi: 10.1038/s42003-025-08923-x, PMID: 41168437 PMC12575609

[137] Bayer A Lennemann NJ Ouyang Y Sadovsky E Sheridan MA Roberts RM . Chromosome 19 microRNAs exert antiviral activity independent from type III interferon signaling. Placenta. (2018) 61:33–8. doi: 10.1016/j.placenta.2017.11.004, PMID: 29277269 PMC5745809

[138] Du Y Wang C Zhang Y . Viral coinfections. Viruses. (2022) 14. doi: 10.3390/v14122645, PMID: 36560647 PMC9784482

[139] Siqueira C Féres V Coutinho L Junqueira I Bento L Montes L . Six cases of Zika/dengue coinfection in a Brazilian cohort, 2015-2019. Viruses. (2020) 12. doi: 10.3390/v12101201, PMID: 33096849 PMC7588971

[140] Dupont-Rouzeyrol M O’Connor O Calvez E Daures M John M Grangeon JP . Co-infection with zika and dengue viruses in 2 patients, New Caledonia, 2014. Emerg Infect Dis. (2015) 21:381–2. doi: 10.3201/eid2102.141553, PMID: 25625687 PMC4313662

[141] Ogwuche J Chang CA Ige O Sagay AS Chaplin B Kahansim ML . Arbovirus surveillance in pregnant women in north-central Nigeria, 2019–2022. J Clin Virol. (2023) 169. doi: 10.1016/j.jcv.2023.105616, PMID: 37944259 PMC10841754

[142] Eligio-García L Crisóstomo-Vázquez MDP Caballero-García M de L Soria-Guerrero M Méndez–galván JF López-Cancino SA . Co-infection of dengue, zika and chikungunya in a group of pregnant women from tuxtla gutiérrez, chiapas: Preliminary data. PLoS Negl Trop Dis. (2020) 14:1–11. doi: 10.1371/journal.pntd.0008880, PMID: 33347432 PMC7785221

[143] João EC Ferreira O da C Gouvêa MI Teixeira M de LB Tanuri A Higa LM . Pregnant women co-infected with HIV and Zika: Outcomes and birth defects in infants according to maternal symptomatology. PLoS One. (2018) 13:e0200168. doi: 10.1371/journal.pone.0200168, PMID: 29979796 PMC6034846

[144] Lynn MK Aquino MSR Rivas PMC Miranda X Torres-Romero DF Cowan H . Perinatal dengue and Zika virus cross-sectional seroprevalence and maternal-fetal outcomes among El Salvadoran women presenting for labor-and-delivery. Matern Health Neonatol Perinatol. (2024) 10:7. doi: 10.1186/s40748-024-00177-5, PMID: 38561854 PMC10985905

[145] Malange VNE Hedermann G Lausten-Thomsen U Hoffmann S Voldstedlund M Aabakke AJM . The perinatal health challenges of emerging and re-emerging infectious diseases: A narrative review. Front Public Health. (2023) 10:1039779. doi: 10.3389/fpubh.2022.1039779, PMID: 36684933 PMC9850110

[146] Aggarwal C Ahmed H Sharma P Reddy ES Nayak K Singla M . Severe disease during both primary and secondary dengue virus infections in pediatric populations. Nat Med. (2024) 30:670–4. doi: 10.1038/s41591-024-02798-x, PMID: 38321219 PMC7617637

[147] Merfeld E Ben-Avi L Kennon M Cerveny KL . Potential mechanisms of Zika-linked microcephaly. Wiley Interdiscip Rev Dev Biol. (2017) 6. doi: 10.1002/wdev.273, PMID: 28383800 PMC5516183

[148] de Araújo TVB Rodrigues LC de Alencar Ximenes RA de Barros Miranda-Filho D Montarroyos UR de Melo APL . Association between Zika virus infection and microcephaly in Brazil, January to May, 2016: preliminary report of a case-control study. Lancet Infect Dis. (2016) 16:1356–63. doi: 10.1016/S1473-3099(16)30318-8, PMID: 27641777 PMC7617035

[149] Campos MC Dombrowski JG Phelan J Marinho CRF Hibberd M Clark TG . Zika might not be acting alone: Using an ecological study approach to investigate potential co-acting risk factors for an unusual pattern of microcephaly in Brazil. PLoS One. (2018) 13:e0201452. doi: 10.1371/journal.pone.0201452, PMID: 30110370 PMC6093667

[150] Carvalho MS Freitas LP Cruz OG Brasil P Bastos LS . Association of past dengue fever epidemics with the risk of Zika microcephaly at the population level in Brazil. Sci Rep. (2020) 10:1752. doi: 10.1038/s41598-020-58407-7, PMID: 32019953 PMC7000767

[151] Thomas SJ . Is new dengue vaccine efficacy data a relief or cause for concern? NPJ Vaccines. (2023) 8. doi: 10.1038/s41541-023-00658-2, PMID: 37061527 PMC10105158

[152] Cracknell Daniels B Ferguson NM Dorigatti I . Efficacy, public health impact and optimal use of the Takeda dengue vaccine. Nat Med. (2025) 31(8):2663–72. doi: 10.1038/s41591-025-03771-y, PMID: 40563017 PMC12353809

